# The therapeutic effects of probiotics on core and associated behavioral symptoms of autism spectrum disorders: a systematic review and meta-analysis

**DOI:** 10.1186/s13034-024-00848-3

**Published:** 2024-12-19

**Authors:** Jen-Chin Lee, Chia-Min Chen, Cheuk-Kwan Sun, I-Ting Tsai, Yu-Shian Cheng, Hsien-Jane Chiu, Ming Yu Wang, Yen-Hsiang Tang, Kuo-Chuan Hung

**Affiliations:** 1 Department of General Psychiatry, Taoyuan Psychiatric Center, Ministry of Health and Welfare, Taoyuan City, Taiwan; 2 Department of Natural Biotechnology, Nanhua University, Chiayi, Taiwan; 3 Department of Emergency Medicine, E-Da Dachang Hospital, I-Shou University, Kaohsiung City, Taiwan; 4 School of Medicine, College of Medicine, I-Shou University, Kaohsiung City, Taiwan; 5 Department of Psychiatry, Tsyr-Huey Mental Hospital, Kaohsiung Jen-Ai’s Home, Kaohsiung City, Taiwan; 6 Institute of Hospital and Health Care Administration, National Yang-Ming Chiao Tung University, Taipei City, Taiwan; 7 Department of Psychiatry, China Medical University Hsinchu Hospital, China Medical University, Hsinchu, Taiwan; 8 Department of Health Services Administration, China Medical University, Taichung, Taiwan; 9 Department of Critical Care Medicine, MacKay Memorial Hospital, Taipei, Taiwan; 10 Department of Medicine, MacKay Medical College, New Taipei City, Taiwan; 11 Department of Anesthesiology, Chi Mei Medical Center, No.901, ChungHwa Road, YungKung Dist, Tainan, 71004 Taiwan

**Keywords:** Probiotics, Autism spectrum disorder, And meta-analysis

## Abstract

**Background:**

We aimed at investigating the efficacies of probiotics in alleviating the core and associated symptoms of autism spectrum disorder (ASD).

**Methods:**

Randomized placebo-controlled trials were identified from major electronic databases from inception to Nov 2023. The outcomes of interests including improvements in the total and associated symptoms of ASD were quantitatively expressed as effect size (ES) based on standardized mean difference (SMD) with 95% confidence interval (CI).

**Results:**

Ten studies with 522 participants (mean age = 8.11) were included in this meta-analysis. The primary results revealed significant improvement in total symptoms in the probiotics group compared with the controls (SMD = − 0.19, *p* = 0.03, ten studies, *n* = 522) but not the core symptoms (i.e., repetitive restricted behaviors, As affiliations 3 and 5 are same, we have deleted the duplicate affiliations and renumbered accordingly. Please check and confirm.problems with social behaviors/communication). Subgroup analyses demonstrated improvement in total symptoms in probiotics users relative to their controls only in studies using multiple-strain probiotics (SMD = − 0.26, *p* = 0.03, five studies, *n* = 288) but not studies using single-strain regimens. Secondary results showed improvement in adaptation (SMD = 0.37, *p* = 0.03, three studies, *n* = 139) and an improvement trend in anxiety symptoms in the probiotics group compared with controls (SMD = − 0.29, 95% CI − 0.60 to 0.02, *p* = 0.07, three studies, *n* = 163) but failed to demonstrate greater improvement in the former regarding symptoms of irritability/aggression, hyperactivity/impulsivity, inattention, and parental stress.

**Conclusions:**

Our study supported probiotics use against the overall behavioral symptoms of ASD, mainly in individuals receiving multiple-strain probiotics as supplements. However, our results showed that probiotics use was only associated with improvement in adaptation and perhaps anxiety, but not core symptoms, highlighting the impact of adaptation on quality of life rather than just the core symptoms. Nevertheless, the limited number of included trials warrants further large-scale clinical investigations.

**Supplementary Information:**

The online version contains supplementary material available at 10.1186/s13034-024-00848-3.

## Introduction

Autism spectrum disorders (ASD) is a group of behavioral manifestations usually with an early onset characterized by restricted and repetitive patterns of interests, behavior, or activities, as well as core behavioral presentations of social and communication issues [1]. ASD is a global health issue with increases in prevalence, incidence, and disability-adjusted life-years (DALYs) from 1990 to 2019 [2], probably due to a change in diagnostic criteria and increase in public awareness [1]. However, current pharmacological treatments mainly focus on the associated behavioral and emotional symptoms of ASD (e.g., irritability or inattention), whereas no pharmacological intervention against the core symptoms has been approved by the U.S. Food and Drug Administration (FDA) [1]. On the other hand, core behavioral expressions of ASD may be viewed as normal manifestations of neurobiological variations within a population and should only be considered symptomatic when interfering with adaptation to daily lives [3]. Therefore, behavioral therapies aimed at developing strategies to reduce distress and functional impairment from the core symptoms of ASD remain the standard treatment [1]. However, limited access to behavioral therapies (e.g., distant location) [4], as well as questionable cost- and time-effectiveness [5] remained significant issues surrounding behavioral therapies. Such a lack of treatment options has contributed to the popularity of complementary and alternative medicine (CAM) among care providers of individuals with ASD, notwithstanding their unclear efficacy [6].

Dietary interventions are among the most popular CAM [6]. The rationale for dietary interventions stems from a proposed link between intestinal microbiota and the behavioral symptoms of ASD through the gut-brain axis (GBA) from previous animal and human studies [7] as well as the frequent gastrointestinal (GI) problems reported in those diagnosed with ASD [8]. In addition, previous experimental studies have shown not only an increase in intestinal mucosal permeability in a mouse model of ASD [9], but also an improvement in such ASD-related abnormal permeability through probiotics administration [10]. Consistently, a number of clinical trials have advocated the use of probiotic-related products as a therapeutic alternative for ASD symptoms [11–19]. Nevertheless, the efficacy of probiotics in this setting remains controversial as significant benefits in the treatment of ASD-related behavioral symptoms relative to placebos were only noted in one study [19], despite the apparently favorable outcomes associated with probiotics in most other studies [11–18]. On the other hand, although probiotics are collectively defined as “live microorganisms which confer a health benefit on the host when administered in adequate amounts”, different formulations of probiotics may contain different strains of microorganism (e.g., *Lactobacillus* and *Bifidobacterium*), as well as different numbers of strains of microorganisms (i.e., single- vs. multi-strain) [20]. Interestingly, although a previous meta-analysis did not demonstrate a significant overall improvement in behavioral outcomes in probiotics users compared to the controls in those diagnosed with ASD, a significant improvement was noted when focusing on studies using probiotics blend compared with the controls on subgroup analysis [21]. In concert with this proposal, a previous experimental investigation showed a potential therapeutic advantage of multi-strain probiotics over single-strain regimens because of a higher chance of favorable microbiota attachment to the intestinal mucosa in the former [22]. Therefore, it is possible that formulations of probiotics may have different effects on symptoms of ASD.

Despite support of the use of probiotic blends against the symptoms of ASD [21], the effectiveness of probiotics in alleviating the core or associated emotional and behavioral symptoms of ASD remained unclear. Indeed, patients diagnosed with ASD frequently present with other behavioral problems (e.g., inattention, irritability, or anxiety) [23], which can be as challenging as the core symptoms to their caregivers [24]. Moreover, previous double-blind placebo-controlled randomized clinical trials have shown the efficacy of probiotics for symptom relief in individuals diagnosed with attention deficit hyperactivity disorder (ADHD) [25, 26] who are frequently comorbid with ASD [27]. Dietary supplementation with probiotics has also been reported to reduce the risk of ASD [28], and even improve neurocognitive functions [29, 30]. Given the anti-inflammatory properties of some probiotics [31, 32], the potential therapeutic effects of probiotics on ASD may be extended to other associated symptoms or comorbidities of ASD rather than confined to the core symptoms of ASD.

Therefore, the current meta-analysis, which included only randomized controlled clinical trials (RCT), aimed at providing reliable updated evidence regarding the efficacies of probiotics in alleviating the core and associated symptoms of ASD. Moreover, the effects of other factors (e.g., number of probiotic strains) on therapeutic outcomes were investigated.

## Methods

### Protocol and registration

This meta-analysis was conducted according to the Preferred Reporting Items for Systematic Reviews and Meta-Analyses (PRISMA) guidelines [33] and registered in the international prospective register of systematic reviews (PROSPERO CRD42023483033).

## Search strategy and selection criteria

Electronic databases, namely PubMed, Cochrane CENTRAL, Embase, and ScienceDirect, were searched for randomized controlled trials (RCTs) that studied the use of probiotics in the treatment of the core or associated symptoms of ASD from inception to November 21, 2023 without restrictions on language and country of origin using appropriate search strategies and keywords (eTable1). The reference lists of the retrieved literature were also scrutinized to avoid missing potentially eligible articles. Criteria pertaining to the population, intervention, comparator, and outcomes (PICO) of the current study were: (1) Population: participants diagnosed with ASD recruited in a RCT, (2) Intervention: probiotics or products using probiotics as a supplement or part of combination therapy, (3) Comparator: non-probiotic interventions or placebo, and (4) Outcome: changes in the core or associated behavioral symptoms of ASD or other related symptoms. On the other hand, studies whose (1) interventions did not include probiotics, (2) design was not RCT, or (3) outcome assessment did not provide data on core or associated behavioral symptoms of ASD were excluded.

## Data extraction and quality assessment

According to the preset keywords and strategies (eTable1), the titles and abstracts of the acquired literature were independently screened by two authors (JC Lee and CM Cheng) who later extracted data on study characteristics and outcomes. Disagreements on study and data eligibility were resolved through discussion with a third author (CK Sun). Inter-rater reliability was evaluated with kappa coefficients [34]. In an attempt to retrieve missing data, the corresponding authors of articles without necessary information were contacted through electronic mails. The quality of a study and the level of evidence for each outcome were appraised with the Cochrane’s “risk of bias” assessment tool [35], and the Grading of Recommendations Assessment, Development, and Evaluation (GRADE) [36], respectively. Discrepancies in opinion between the two authors about the risk of bias or certainty of evidence ratings were settled by discussion.

## Data synthesis and analysis

Primary outcomes of the present study were changes in the core symptoms of ASD, including overall behavioral symptoms, social behaviors, restricted repetitive behaviors (RRB), and communication, assessed with standardized rating scales or assessment tools such as the autism diagnostic observation schedule (ADOS), the Achenbach system of empirically based assessment (ASEBA), the clinical global impression– severity (CGI-S), the aberrant behavior checklist (ABC), and the social responsiveness scale (SRS). Secondary outcomes included all behavioral symptoms or issues associated with ASD such as inattention, hyperactivity/impulsivity, irritability, anxiety, adaptation, and parental stress. The outcomes of interest were quantitatively expressed as effect sizes (ES), which were presented as standardized mean differences (SMD) with 95% confidence intervals (CI) for continuous variables. Review Manager 5 (RevMan5.4; Copenhagen: The Nordic Cochrane Center, The Cochrane Collaboration,2014) was adopted to conduct all data analyses. For the analysis of continuous data, the generic inverse-variance approach was used. To examine the robustness of study outcomes, the impact of individual studies was evaluated with sensitivity analysis using a leave-one-out approach. Heterogeneity and the probability of publication bias of the included studies were assessed with *I*-squared test and funnel plot inspection, respectively. Subgroup analyses were conducted focusing on the therapeutic strategies (supplementation vs. combination) and number of microbiome strains in probiotics (single- vs. multiple-strain) to scrutinize the possible effects on treatment outcomes. A *p* value of under 0.05 was deemed statistically significant for all study outcomes.

## Results

### Eligible studies and characteristics

**Fig. 1:**
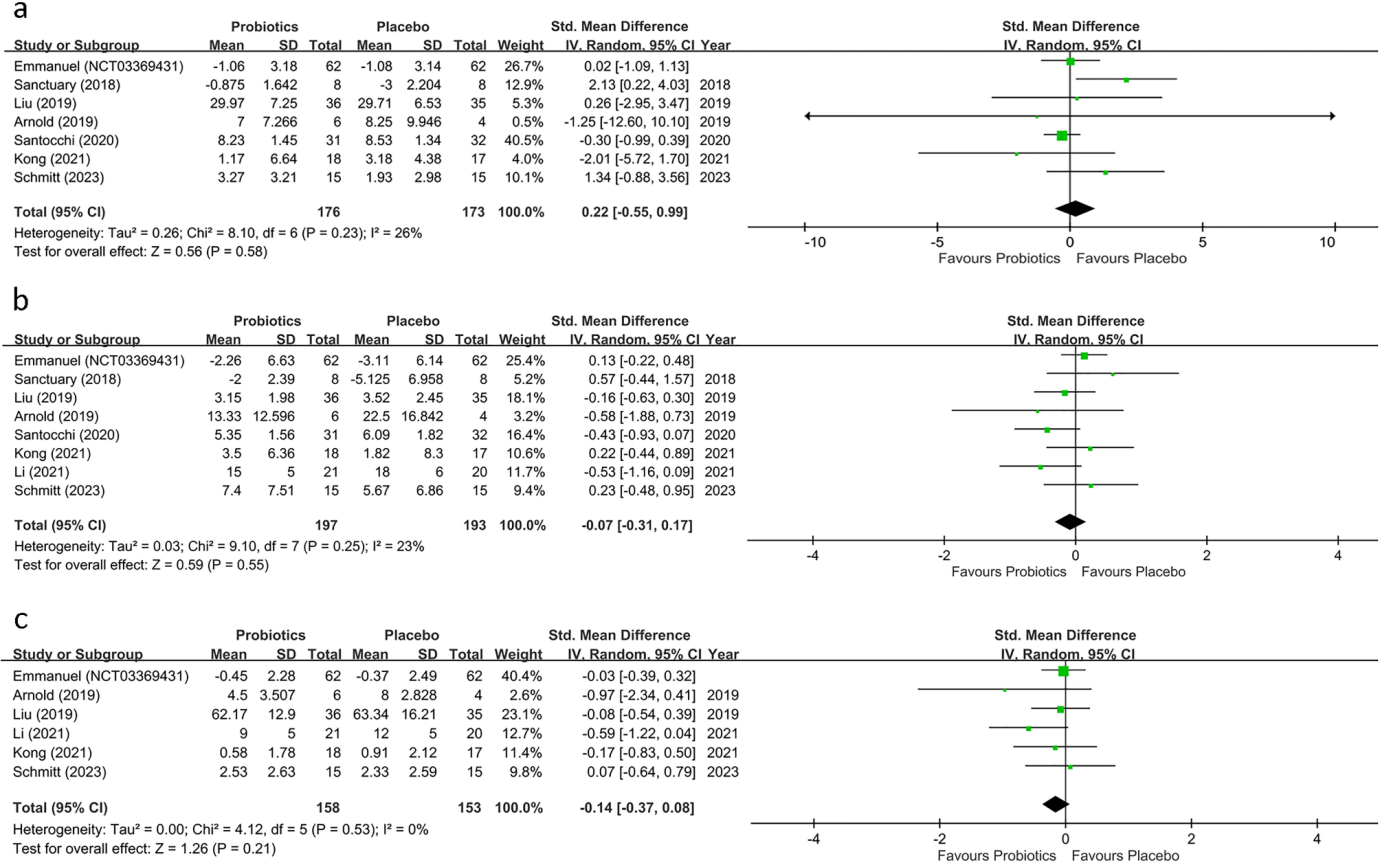
PRISMA diagram of identifying eligible studies. *ASD* autism spectrum disorder

In accordance with the PRISMA statement [33] (Fig. 1)0.336 articles were initially identified from the electronic databases using predetermined search strategies (eTable2). Following the exclusion of 312 studies through title and abstract screening, 24 were subjected to full-text review that finally yielded ten eligible studies including 522 participants [11–18, 37, 38]. (Fig. 1) with a kappa coefficient for study eligibility being 1. Information from the eligible studies was extracted on November 23, 2023. All ten studies recruited children or adolescents except one that enrolled young adults with age ranging from 15 to 45 years with a mean age of 20 [18]. (Table 1). The mean age of the participants from the ten studies was 8.11 years (SD = 4.86). In respect of the number of probiotic strains, five trials used multiple-strain probiotics [11–13, 17, 38] and five used single-strain regimens [14–16, 18, 38]. The median duration of treatment was ten weeks with a range of 4–24 weeks. The use of single or multiple psychotropics was allowed in most studies regardless of their nature with the exception of one trial that excluded participants under psychotropic treatment other than methylphenidate [15]. Regarding study design, six trials used a parallel design [13–17, 38] with the other four being cross-over studies [11, 12, 18, 37]. While eight studies administered probiotics as diet supplements, one combined probiotics with behavioral intervention (i.e., applied behavioral analysis) [38] and the other used probiotics with another dietary supplement (e.g., bovine colostrum) [37]. The countries of origin of the included studies were USA [14, 17, 18, 37], Taiwan [15, 16], Italy [13, 17], China [38] and the UK [11]. With regard to adverse events, except two studies that did not provide relevant information [13, 38],, all other trials showed that probiotics were well-tolerated and associated with either no or very mild side effects such as increased gassiness or loose stools [11, 12, 14–18, 37], without any serious adverse events being reported [11, 12, 14–18, 37].

**Table 1:** Summary of characteristics of studies in the current meta-analysis

Study (year)	Diagnosis (Criteria)	Design	Comparison	*N*	Duration (weeks)	Outcome	Psychotropic medications	Mean age (years)	Female (%)	Country
Billeci (2023)	ASD (DSM-5)	RCT	Probiotics: multiple strains	20	24	1.Overall = ADOS CSS 2.Adaptation = VABS-II	Allow	3.88 (1.5–6)	23.9	Italy
			Placebo	26						
Liu (2023)	ASD (DSM-5)	RCT	Probiotics: single strain	41	8	1.Overall = ASEBA 2.Irritability = ASEBA aggression 4.Hyperactivity = ADHDT 5.Inattention = ADHDT 6.Anxiety = ASEBA anxiety	Allow only psychostimulant	4.82 (2.5–7)	12.2	Taiwan
			Placebo	41						
Schmitt (2023)	ASD (DSM-5)	RCT/ Crossover	Probiotics: single strain	15	4	1.Overall = CGI-S 2.RRB = ABC Stereotype 3.Social = ABC lethargy 4.Communication = ABC inappropriate speech 5.Irritability = ABC Irritability 6.Hyperactivity = ABC Hyperactivity 7.Adaptation = Vineland adaptive behavior composite score	Allow	20 (15–45)	0	USA
			Placebo	15						
Kong (2021)	ASD (DSM-IV TR/−5)	RCT	Probiotics: single strain	18	16	1.Overall = ABC total 2.RRB = ABC Stereotype 3.Social = ABC lethargy 4.Communication = ABC inappropriate speech 5.Irritability = ABC Irritability 6.Hyperactivity = ABC Hyperactivity	Allow	10.26 (3–25)	25.8	USA
			Placebo	17						
Li (2021)	ASD (DSM-5)	RCT	Probiotics: multiple strains + ABA	21	12	1.Total = ATEC 2.Social = ATEC social behaviors 3.Communication = ATEC communication	Allow	4.55 (3–6)	24.4	China
			ABA only	20						
Santocchi (2020)	ASD (DSM-5)	RCT	Probiotics: multiple strains	6	24	1.Total = ADOS CSS 2.Social = ADOS Social affect 3.RRB = ADOS RRB 4.Adaptation = VABS-II 5.Parental stress = PSI	Allow	4.14 (1.5–6)	16.4	Italy
			Placebo	4						
Arnold (2019)	ASD (DSM-5)	RCT/ Crossover	Probiotics: multiple strains	31	8	1.Overall = SRS total 2.RRB = ABC Stereotype 3.Social = ABC lethargy 4.Communication = ABC inappropriate speech 5.Irritability = ABC Irritability 6.Hyperactivity = ABC Hyperactivity 7.Parental stress = PSI 8.Anxiety = PRAS-ASD total	Allow	8.85 (3–12)	40	USA
			Placebo	32						
Liu (2019)	ASD (DSM-5)	RCT	Probiotics: single strain	36	4	1.Overall = ABC-T total 2.RRB = SRS autism mannerism 3.Social = ABC-T social and self-help 4.Communication = SRS social communication 5.Irritability = CBCL aggressive behaviors 6.Hyperactivity = SNAP-IV 7. Inattention = SNAP-IV 8.Anxiety = CBCL anxiety	Allow	10.01 (7–15)	0	Taiwan
			Placebo	35						
Sanctuary (2018)	ASD (ADOS)	RCT/ Crossover	Probiotics: single strain + BCP	8	5	1.Overal = ABC total score 2.RRB = ABC Stereotype 3.Social = ABC lethargy 4.Irritability = ABC Irritability 5.Hyperactivity = ABC Hyperactivity	Allow	6.8 (2–11)	12.5	USA
			BCP	8						
Emmanuel NCT03369431	ASD (ADI-R, DISCO, ADOS)	RCT/ Crossover	Probiotics: multiple strains	64	12	1.Overall = ATEC total 2.RRB = ABC Stereotype 3.Social = ABC lethargy 4.Irritability = ABC Irritability 5.Hyperactivity = ABC Hyperactivity 6.Communication = ABC inappropriate speech 7.Parental stress = APSI	Allow	7.8 (3–16)	17.4	UK
			Placebo	64						

*ABA* applied behavior analysis; *ABC* aberrant behavior checklist; *ABC-T* aberrant behavior checklist-Taiwan version; *ADHDT* attention-deficit/hyperactivity disorder test; *ADI-R* autism diagnostic interview-revised; *ADOS* autism diagnostic observation schedule; *ADOS CSS* autism diagnostic observation schedule calibrated severity score; *APSI* autism parenting stress index; *ASD* autistic spectrum disorder; *ASEBA* Achenbach system of empirically based assessment; *ATEC* autism treatment evaluation checklist; *CBCL* child behavior checklist; *CGI-S* clinical global impressions scale– severity; *BCP* bovine colostrum product; *DISCO* diagnostic interview for social and communication disorders; *DSM-IV-TR* diagnostic and statistical manual of mental disorders, fourth edition, text revision; *DSM-5* diagnostic and statistical manual of mental disorders fifth edition; *N* number; *PRAS-ASD* total parent-rated anxiety scale for ASD; *PSI* parenting stress index; *RCT* randomized controlled trial; *RRB* restricted repetitive behaviors; *SNAP-IV* Swanson, Nolan and Pelham (SNAP)-IV-Taiwan version; *SRS* social reporting standard; VABS-II Vineland Adaptive Behavior Scales-II

## Risk of bias appraisal

**Fig. 2:**
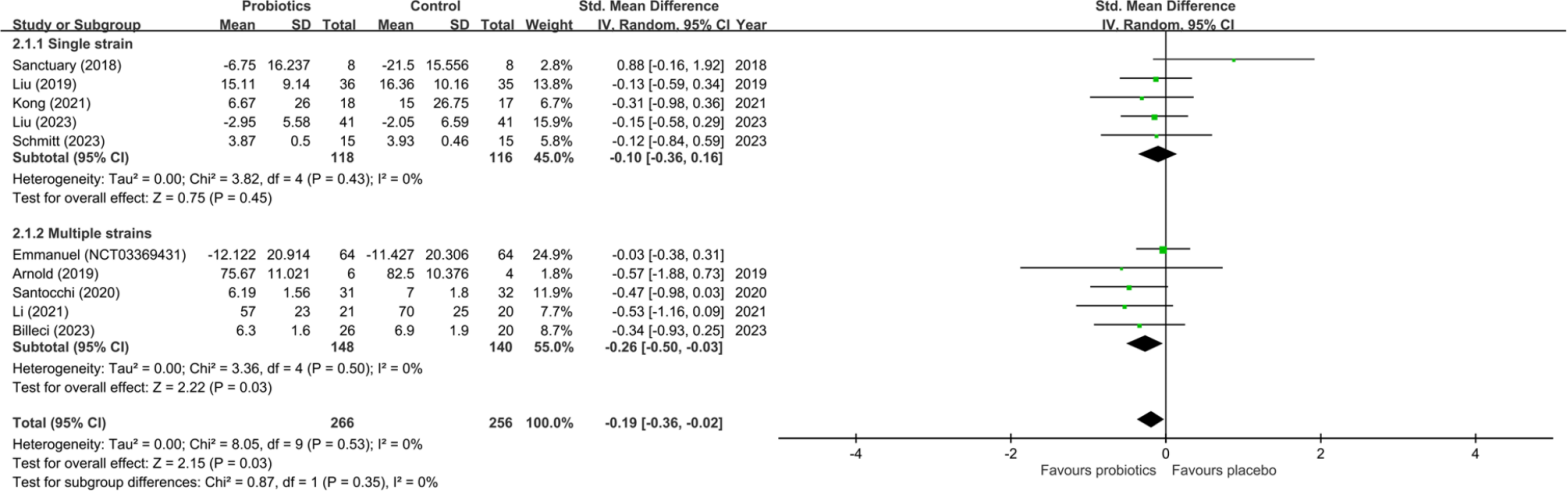
Risk of bias for eligible studies. *Study retrieved from clinicaltrials.gov. ^X^Sponsored by pharmaceutical company.

Risk of bias assessment with the Cochrane Collaboration’s tool indicated a low risk of bias in allocation concealment and randomization sequence in the majority of studies. Similarly, with the exception of two trials that did not recruit placebo controls [37, 38], detection and performance biases were considered low in other trials due to their double-blind design. Sensitivity analysis demonstrated no significant impact of the two studies [37, 38] on the primary outcome (SMD=−0.19, 95%CI:−0.38~−0.01, *p* = 0.04). Relatively long follow-up periods in another two studies contributed to their high risk of attrition bias [13, 14]. (Fig. 2). With regard to reporting bias, it was considered low in all studies because all of them chose behavioral issues of ASD as the primary outcomes (Fig. 2). Nevertheless, a high risk of other bias was assigned to three studies due to sponsorship from private companies [15, 16, 18] (Fig. 2).

## Primary outcome

**Fig. 3:**
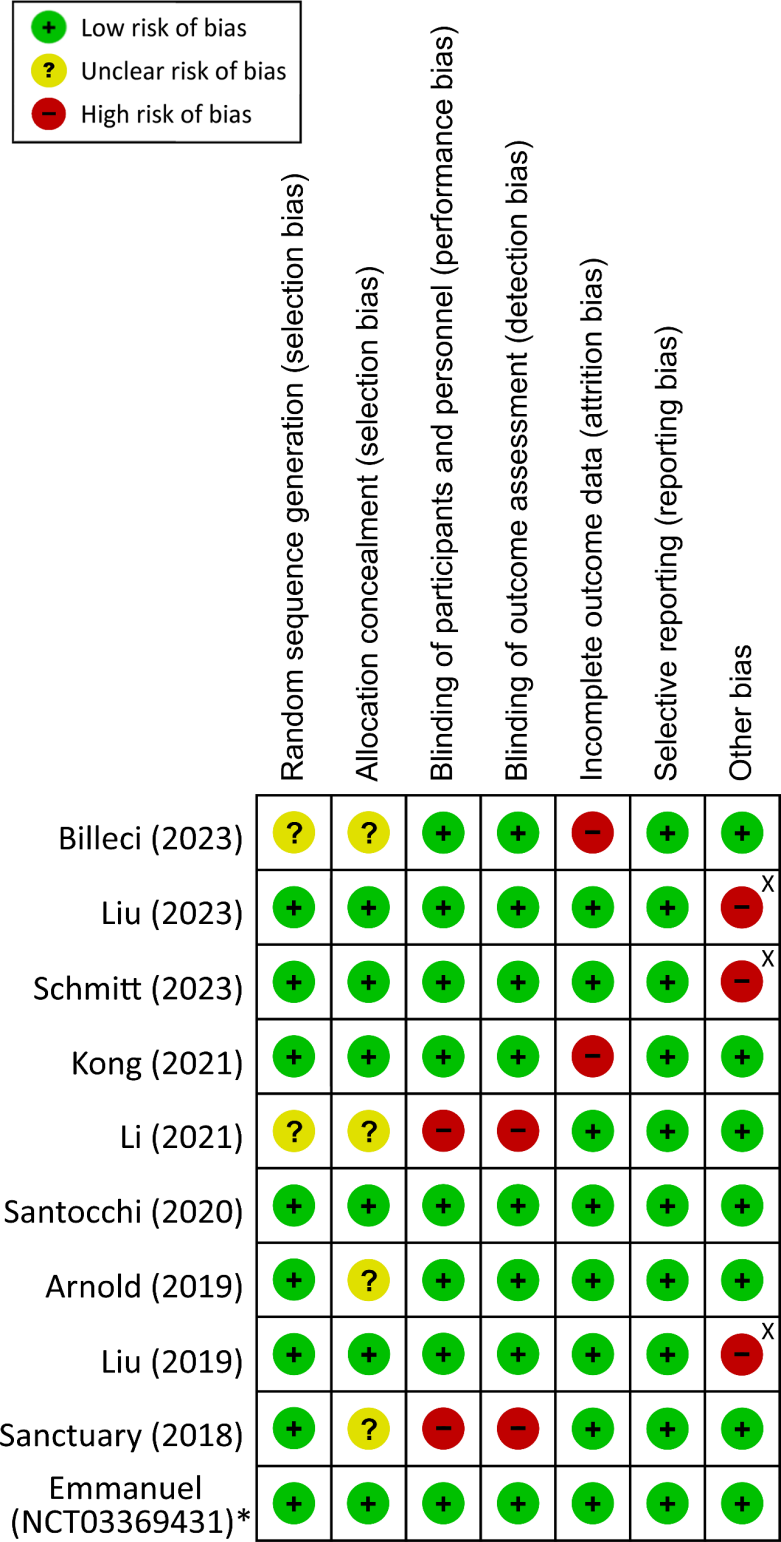
Forest plot of effect size for comparing the difference in the overall behavioral symptoms of autism spectrum disorder between probiotics and control groups with subgroups comparison between single-strained and multiple-strained probiotics. *CI* confidence interval; *Std* standardized; *SE* standard error

**Fig. 4:**
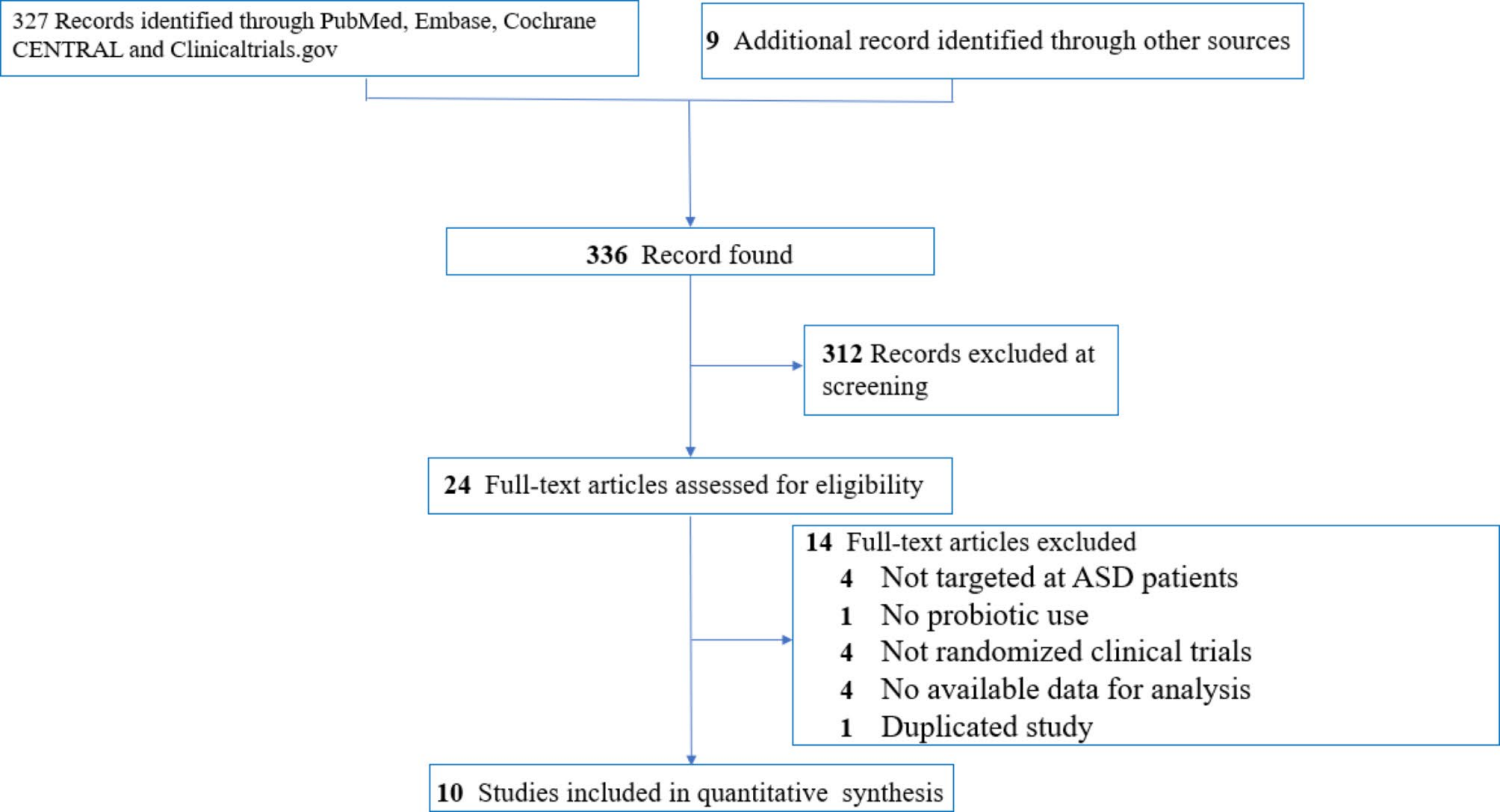
**a** Forest plot of effect size for comparing the difference between probiotics and control groups for the restricted repetitive behaviors; **b** social behaviors; **c** communication. *ASD* autism spectrum disorder; *CI* confidence interval; *Std* standardized; *SE* standard error

The current study revealed a significant improvement in the overall behavioral symptoms of ASD in individuals receiving probiotics compared with the controls (SMD=−0.19, 95%CI:−0.36~−0.02, *p* = 0.03, ten studies with 522 participants) (Fig. 3). Heterogeneity across the included studies was non-significant (*I*^22^ = 0% and *p* = 0.53). Besides, funnel plot inspection demonstrated no notable asymmetry for the primary outcome (eFigure1). On the other hand, sensitivity analysis showed a loss of significance regarding the primary outcome in the probiotics group when either one of three studies was excluded [13, 17, 38], although the results were still in favor of probiotics use. Subgroup analyses supported an association between a significant improvement in the total symptoms of ASD and the use of multiple-strain probiotics relative to their controls (SMD = − 0.26, 95% CI − 0.50~ − 0.03, *p* = 0.03, five studies with 288 participants) but not when comparing between single-strain regimen group and the control group (SMD = − 0.16, 95% CI − 0.36 ~ 0.16, *p* = 0.45, five studies with 234 participants) (Fig. 3). However, a direct comparison between the subgroup of studies using multiple-strain probiotics and that adopting single-strain products showed no significant difference (SMD = 0.26 v.s − 0.16, *p* = 0.35) (Fig. 3). Our subgroup analysis focusing on the choice of probiotics administration strategies on treatment outcome demonstrated significant therapeutic benefits in studies adopting probiotics as supplements when compared to the controls (SMD = − 0.19, 95%CI − 0.38~−0.01, *p* = 0.03, eight studies with 465 participants) (eFigure2). With regard to the core symptoms of ASD, the current study showed no significant improvement in ASD-associated RRB, social behaviors, or communication problems in the probiotics group compared to the control group (SMD = 0.22, 95% CI − 0.55 ~ 0.99, *p* = 0.58, seven studies with 349 participants, SMD = − 0.07, 95%CI − 0.31 ~ 0.17, *p* = 0.55, eight studies with 390 participants, SMD − 0.14, 95% CI − 0.37 ~ 0.08, *p* = 0.21, six studies with 311 participants, respectively) (Fig. 4a, b and c). No significant heterogeneity was noted for RRB (*I*^22^ = 26% and *p* = 0.23), social behaviors (*I*^22^ = 23% and *p* = 0.25), and communication (*I*^22^ = 0% and *p* = 0.53). In addition, the results demonstrated neither inconsistency on leave-one-out sensitivity analysis nor notable asymmetry on funnel plot inspection for the outcomes of RRB, social behaviors, and communication (eFigure3-5).

### Secondary outcomes

Our analysis showed no significant difference in the associated problems of ASD between the probiotics and control groups in terms of irritability/aggression (eFigure6), hyperactivity/impulsivity (eFigure7), parental stress (eFigure8), and inattention (eFigure9). Besides, no significant heterogeneity, inconsistency on leave-one-out sensitivity analysis, or notable asymmetry on funnel plot inspection was found for the symptoms of irritability/aggression, hyperactivity/impulsivity, parental stress and inattention (eFigure10-13). Despite the lack of statistical significance, there was a trend of greater improvement in symptoms of anxiety in the probiotics group than that in the control groups (SMD=−0.29, 95%CI:−0.60 ~ 0.02, *p* = 0.07, three studies with 163 participants) (eFigure14). No significant heterogeneity or asymmetry on funnel plot inspection (eFigure15) was discernible. Sensitivity analysis with the leave-one-out approach showed a significantly higher degree of improvement in the symptoms of anxiety in the probiotics group than that in the control group after excluding the study by Liu et al. [16]. Moreover, our findings revealed a significantly greater improvement in adaptation mainly assessed with the vineland adaptive behavior scales in the probiotics group than that in the control groups (SMD = 0.37, 95%CI:0.03 ~ 0.71, *p* = 0.03, three studies with 139 participants) (eFigure16) without significant heterogeneity or asymmetry on funnel plot inspection (eFigure17).

### Certainty of evidence

Details regarding the certainty of evidence for individual outcomes according to the GRADE guidelines are summarized in eTable3. The evidence of our primary outcomes focusing on improvement in the overall behavioral symptoms of ASD, RRB and social behaviors of ASD were downgraded to moderate because of the limited number of eligible studies. Our primary outcome pertinent to improvement in communication was further downgraded to low on account of even more limited data availability. In respect of secondary outcomes, the certainty of evidence regarding irritability/aggression and hyperactivity/impulsivity was downgraded to low due to the limited number of eligible trials and the fact that such outcomes were not direct targets of those studies. With regard to the secondary outcomes of parental stress, inattention, anxiety, and adaptation, the level of evidence was downgraded to very low on the ground of notably limited data availability for a precise analysis.

## Discussion

To our best knowledge, this is the first meta-analysis that investigated the effects of probiotics on the core symptoms as well as the behavioral and emotional problems of ASD. A previous meta-analysis, which only focused on the overall behavioral symptoms of ASD, failed to show significant improvement in subjects with ASD treated with probiotics compared to the control group [21]. By contrast, the results of the present meta-analysis, which included ten RCTs with 522 participants, demonstrated significant improvement in the overall behavioral symptoms in the probiotics group compared with the control group but without notable beneficial impact of probiotics on the core symptoms of ASD including social interaction, communication, and RRB. Our secondary analyses further showed significant improvement in the adapted behaviors in the probiotics groups compared to the controls as well as a non-significant trend of probiotics-related improvement in anxiety symptoms. On the other hand, no significant difference was noted in other secondary outcomes including irritability/aggression, hyperactivity/impulsivity, inattention, and parental stress between the two groups. Overall, our study supported an association of probiotics use with improvement in the behavioral but not the core symptoms of ASD. Besides, the use of probiotics correlated with significantly improved adapted behaviors but only a trend of improvement in anxiety.

In contrast to our finding of significant improvement in the overall behavioral symptoms of ASD in the probiotics group compared to the controls, a previous meta-analysis did not show a significant difference between the two groups despite demonstration of a trend in favor of the former [21]. The inclusion of up to ten RCTs in our investigation compared to seven studies in the previous meta-analysis [21] may contribute to the difference in findings. Several mechanisms may explain the association between probiotics use and the observed alleviation of ASD symptoms [7]. One of the key hypotheses involves the gut-brain-axis involving a network of neuroendocrine pathways that enable a bidirectional communication between intestinal microbiome and the central nervous system [7]. Consistently, a previous study has identified the vagus nerve as the route of communication between intestinal microbiome and the central gamma-aminobutyric acid system [39]. Moreover, given the known association between systemic inflammation and neurocognitive impairment [40, 41], the anti-inflammatory properties of certain probiotics [31, 32] may be beneficial to the maintenance of neurocognitive functions. Besides, a prior experimental study using a mouse ASD model has demonstrated a correlation of an abnormal increase in intestinal mucosal permeability (e.g., dysbiosis) with systemic inflammation and abnormal neurotransmitter signaling in the brain [42]. Adopting the same ASD model, another study further showed a normalization of such an increased mucosal permeability through the oral administration of the probiotic *B. fragilis* [10]. Nevertheless, although our results are supported by prior studies that suggested a possible modulating role of probiotics in GBA which has been reported to be associated with the behavioral symptoms of ASD, evidence derived from the current study is still not solid enough given the limited number of available trials.

Consistent with the results of a previous meta-analysis that showed significant improvements in the overall behavioral symptoms in subjects diagnosed with ASD treated with probiotic blends compared with controls [21], our subgroup analysis focusing on the use of single- versus multiple-strain probiotics demonstrated a significant improvement in the overall symptoms of ASD only in the multiple-strain probiotics group compared to the control group. Consistently, a prior animal study suggested the merit of multiple-strain probiotics due to an increased opportunity of adhesion of beneficial microbiota to intestinal mucosa [22]. Additionally, another meta-analysis reported the effectiveness of using multiple- instead of single-strain probiotics in the prevention of necrotizing enterocolitis and mortality in preterm infants [43]. Taken together, our findings and those from a previous meta-analysis [21] supported the use of multiple-strain probiotics for the alleviation of ASD symptoms. Nevertheless, the fact that only five studies were available for subgroup analysis suggests the need for further large-scale studies to address this issue.

Because most RCTs in our study used probiotics as dietary supplements except two that combined probiotics with other therapeutic approaches (i.e., probiotics plus bovine colostrum product or probiotics plus applied behavior analysis) [37, 38], we conducted subgroup analysis focusing on studies using probiotics only as supplements that consistently showed greater improvement in the overall behavioral symptoms of ASD in probiotics users than that in the controls. The results, therefore, suggested that probiotic supplementation without combining with other treatments may be effective in this setting, although our findings await further validation due to the limited number of RCTs and the small ES.

Despite the significant overall behavioral improvement in subjects diagnosed with ASD treated with probiotics compared with the controls, no significant difference in improvement was noted in the three core symptoms of ASD (i.e., social functioning, communication, and RRB) between the two groups. There are several possible reasons for this observation. First, the overall improvement in behavioral symptoms may be attributed to the collective minor improvements in each core domain of behavioral problems. However, statistically significant differences may be obscured by the small number of RCTs available for analyzing each core behavioral symptom. Second, the lack of available information about changes in the core behavioral symptoms in two RCTs that provided findings in favor of probiotics in the treatment of the overall symptoms of ASD [13, 38] may render the improvement in core symptoms non-significant because of the absence of their probable positive contributions. Third, the observed behavioral improvement may stem from other associated symptoms of ASD such as mood or irritability. In summary, our study could not provide robust evidence either to support or dismiss the effectiveness of probiotics for each core behavioral symptom of ASD due to the small numbers of available trials (i.e., a maximum of eight RCTs for each core behavioral symptom). Future studies are warranted to elucidate this issue.

Despite the lack of significant probiotics-associated improvement in the core behavioral symptoms of ASD, our results on secondary outcomes showed a significant improvement in adaptive behaviors assessed mainly by the vineland adaptive behavior scales in subjects treated with probiotics compared with controls. Consistently, prior RCTs have reported an association between the use of probiotics and cognitive function improvement [44, 45], which may be attributed to the systemic anti-inflammatory properties of certain probiotics [40, 41], that may be protective against neurocognitive impairment given the known negative effect of systemic inflammation on neurocognitive functions [31, 32]. Although we were unable to evaluate the result of cognitive function with standardized tools such as Wechsler Intelligence Scale for Children (WISC) due to a lack of available data, our finding of improved adaptive functions may imply a beneficial influence of probiotics on overall adaptation. However, the limited number of available RCTs (*n* = 3) suggests the need for further studies based on more objective cognitive assessment (e.g., WISC) to verify our findings.

Analyses of our secondary outcomes focusing on other associated problems of ASD showed an apparent trend of improvement in anxiety symptoms in individuals treated with probiotics compared with the controls despite a lack of statistical significance, while no difference was noted in other behavioral issues of ASD including irritability/aggression, inattention, hyperactivity/impulsivity, and parental stress between the two groups. Previous research has demonstrated a correlation between intestinal microbiome and mood regulation [46]. Compared with the intestinal microbiota composition in healthy individuals, prior investigations have also shown increased levels of the phyla *Bacteroidetes*, *Proteobacteria*, and *Actinobacteria* but a reduced level of *Firmicutes* in those diagnosed with major depressive disorders [47]. One proposed hypothesis is the mood-modulatory effect of probiotics through the microbial-gut-brain axis [48]. Despite the reported superiority of probiotics over placebos in anxiety relief from previous RCTs [49, 50], a recent meta-analysis showed effectiveness of probiotics only against depressive symptoms but not anxiety in patients with anxiety- or depression-related diagnoses [51]. Since one out of the three included RCTs used a measurement tool for both depression and anxiety [15] but the remaining two used tools for only anxiety [12, 16], the mixed results from both depression and anxiety may influence the specificity of our outcomes. Nevertheless, given our finding of a nearly significant effect and the positive results of a previous meta-analysis for mood symptoms [51], more investigations into the efficacy of probiotics in improving mood symptoms specifically targeting anxiety or depression in patients with ASD are warranted.

Several limitations in this study need to be taken into consideration. First, one of the major limitations of the current study is that most of our included trials did not investigate the effect of probiotics on GI symptoms. For instance, chronic constipation, which is noted in a significant number of children diagnosed with ASD and can adversely affect their quality of life [52], was not specified in the included studies. Taking into account the therapeutic potential of probiotics against chronic constipation [53], it is difficult to determine whether the observed improvement in symptoms of ASD was associated with alleviation of GI symptoms (e.g., constipation). Therefore, we strongly recommend the survey of changes in GI symptoms in future probiotics-related trials to rule out the potential confounding effects in this clinical setting. Second, notwithstanding our inclusion of up to ten trials and a total of 522 participants, the results were still not robust enough to provide solid evidence. Moreover, our results on some core or associated symptoms of ASD, which were derived from limited numbers of available trials (i.e., only two trials for inattention), require validation from future studies. Third, certain heterogeneities in treatment strategies such as probiotics used as supplements or part of combination therapy may be potential confounding factors that influence the therapeutic outcomes. Nevertheless, our consistent findings on subgroup analysis after excluding studies using probiotics as part of combination therapies indicated minimal influence of this confounder. Forth, a number of other factors that may affect the therapeutic outcomes of probiotics (e.g., dietary habits, use of other nutritional supplements) were unaccounted for due to a lack of relevant information for meta-regression or subgroup analysis. Finally, two studies that did not use placebo control may be more susceptible to performance and detection bias [37, 38]. Nevertheless, our sensitivity test demonstrated consistent results after excluding those two studies.

## Conclusions

The current study showed a significantly greater improvement in the overall behavioral symptoms of ASD in participants treated with probiotics than that in the control groups. Our subgroup analyses further demonstrated a significant alleviation of the behavioral symptoms of ASD in those receiving multiple-strain probiotics compared to controls. Moreover, except a significant improvement in adaptation and an apparent trend of improvement in mood symptoms, the use of probiotics was not associated with significant mitigation of core or other associated symptoms of ASD. Nevertheless, all double blinded placebo-controlled studies failed to address probiotics-related improvement in gastrointestinal (GI) symptoms (e.g., chronic constipation) that may be a significant confounder in the assessment of study outcome. Our results, which were derived from a limited number of available trials that provided limited information on changes in GI symptoms warrant further large-scale clinical investigations to shed light on the mechanisms underlying the observed improvement in the total symptoms of ASD and verify our findings.

## Electronic supplementary material

Supplementary Material 1

## Data Availability

No datasets were generated or analysed during the current study.

## References

[1] Hirota T, King BH. Autism spectrum disorder: a review. JAMA. 2023;329(2):157–68. 36625807 10.1001/jama.2022.23661

[2] Li YA, Chen ZJ, Li XD, Gu MH, Xia N, Gong C, Zhou ZW, Yasin G, Xie HY, Wei XP, et al. Epidemiology of autism spectrum disorders: global burden of disease 2019 and bibliometric analysis of risk factors. Front Pediatr. 2022;10:972809. 36545666 10.3389/fped.2022.972809 PMC9760802

[3] Megari K, Frantzezou CK, Polyzopoulou ZA, Tzouni SK. Neurocognitive features in childhood & adulthood in autism spectrum disorder: a neurodiversity approach. Int J Dev Neuroscience: Official J Int Soc Dev Neurosci 2024. 10.1002/jdn.10356 38953464

[4] Littman ER, Gavin L, Broda A, Hodges AC, Spector L. Barriers to receiving applied behavior analysis services in children with autism spectrum disorder. Cureus. 2023;15(11):e48585. 38084161 10.7759/cureus.48585 PMC10710535

[5] Hodgson R, Biswas M, Palmer S, Marshall D, Rodgers M, Stewart L, Simmonds M, Rai D, Le Couteur A. Intensive behavioural interventions based on applied behaviour analysis (ABA) for young children with autism: a cost-effectiveness analysis. PLoS ONE. 2022;17(8):e0270833. 35972929 10.1371/journal.pone.0270833 PMC9380934

[6] Brondino N, Fusar-Poli L, Rocchetti M, Provenzani U, Barale F, Politi P. Complementary and alternative therapies for autism spectrum disorder. Evid Based Complement Alternat Med. 2015;2015:258589. 26064157 10.1155/2015/258589 PMC4439475

[7] Morton JT, Jin D-M, Mills RH, Shao Y, Rahman G, McDonald D, Zhu Q, Balaban M, Jiang Y, Cantrell K, et al. Multi-level analysis of the gut–brain axis shows autism spectrum disorder-associated molecular and microbial profiles. Nat Neurosci. 2023;26(7):1208–17. 37365313 10.1038/s41593-023-01361-0 PMC10322709

[8] Madra M, Ringel R, Margolis KG. Gastrointestinal issues and Autism Spectrum Disorder. Child Adolesc Psychiatr Clin N Am. 2020;29(3):501–13. 32471598 10.1016/j.chc.2020.02.005 PMC8608248

[9] Fattorusso A, Di Genova L, Dell’Isola GB, Mencaroni E, Esposito S. Autism Spectrum disorders and the gut microbiota. Nutrients 2019, 11(3). 10.3390/nu11030521 PMC6471505 30823414

[10] Hsiao EY, McBride SW, Hsien S, Sharon G, Hyde ER, McCue T, Codelli JA, Chow J, Reisman SE, Petrosino JF, et al. Microbiota modulate behavioral and physiological abnormalities associated with neurodevelopmental disorders. Cell. 2013;155(7):1451–63. 24315484 10.1016/j.cell.2013.11.024 PMC3897394

[11] Anton V, Emmanuel M. FRCP: Efficacy of vivomixx on behaviour and gut function in autism spectrum disorder (VIVO-ASD) (NCT03369431). In.; 2022-03–8.

[12] Arnold LE, Luna RA, Williams K, Chan J, Parker RA, Wu Q, Hollway JA, Jeffs A, Lu F, Coury DL, et al. Probiotics for gastrointestinal symptoms and quality of life in Autism: a placebo-controlled pilot trial. J Child Adolesc Psychopharmacol. 2019;29(9):659–69. 31478755 10.1089/cap.2018.0156 PMC7364307

[13] Billeci L, Callara AL, Guiducci L, Prosperi M, Morales MA, Calderoni S, Muratori F, Santocchi E. A randomized controlled trial into the effects of probiotics on electroencephalography in preschoolers with autism. Autism. 2023;27(1):117–32. 35362336 10.1177/13623613221082710 PMC9806478

[14] Kong XJ, Liu J, Liu K, Koh M, Sherman H, Liu S, Tian R, Sukijthamapan P, Wang J, Fong M et al. Probiotic and oxytocin combination therapy in patients with autism spectrum disorder: a randomized, double-Blinded, placebo-controlled pilot trial. Nutrients 2021, 13(5). 10.3390/nu13051552 PMC8147925 34062986

[15] Liu Y-W, Wang J-E, Sun F-J, Huang Y-H, Chen H-J. Probiotic intervention in young children with autism spectrum disorder in Taiwan: a randomized, double-blinded, placebo-controlled trial. Res Autism Spectr Disorders. 2023;109:102256.

[16] Liu YW, Liong MT, Chung YE, Huang HY, Peng WS, Cheng YF, Lin YS, Wu YY, Tsai YC. Effects of Lactobacillus plantarum PS128 on children with Autism Spectrum Disorder in Taiwan: a Randomized, Double-Blind, placebo-controlled trial. Nutrients 2019, 11(4). 10.3390/nu11040820 PMC6521002 30979038

[17] Santocchi E, Guiducci L, Prosperi M, Calderoni S, Gaggini M, Apicella F, Tancredi R, Billeci L, Mastromarino P, Grossi E, et al. Effects of Probiotic supplementation on gastrointestinal, sensory and core symptoms in Autism Spectrum disorders: a Randomized Controlled Trial. Front Psychiatry. 2020;11:550593. 33101079 10.3389/fpsyt.2020.550593 PMC7546872

[18] Schmitt LM, Smith EG, Pedapati EV, Horn PS, Will M, Lamy M, Barber L, Trebley J, Meyer K, Heiman M, et al. Results of a phase ib study of SB-121, an investigational probiotic formulation, a randomized controlled trial in participants with autism spectrum disorder. Sci Rep. 2023;13(1):5192. 36997569 10.1038/s41598-023-30909-0 PMC10061375

[19] Wang Y, Li N, Yang JJ, Zhao DM, Chen B, Zhang GQ, Chen S, Cao RF, Yu H, Zhao CY, et al. Probiotics and fructo-oligosaccharide intervention modulate the microbiota-gut brain axis to improve autism spectrum reducing also the hyper-serotonergic state and the dopamine metabolism disorder. Pharmacol Res. 2020;157:104784. 32305492 10.1016/j.phrs.2020.104784

[20] Picard C, Fioramonti J, Francois A, Robinson T, Neant F, Matuchansky C. Review article: bifidobacteria as probiotic agents -- physiological effects and clinical benefits. Aliment Pharmacol Ther. 2005;22(6):495–512. 16167966 10.1111/j.1365-2036.2005.02615.x

[21] He X, Liu W, Tang F, Chen X, Song G. Effects of Probiotics on Autism Spectrum disorder in children: a systematic review and Meta-analysis of clinical trials. Nutrients 2023, 15(6). 10.3390/nu15061415 PMC10054498 36986145

[22] Collado MC, Meriluoto J, Salminen S. In vitro analysis of probiotic strain combinations to inhibit pathogen adhesion to human intestinal mucus. Food Res Int. 2007;40(5):629–36.

[23] Pandolfi V, Magyar CI. Assessment of Co-occurring Emotional and Behavioral Disorders in Youth with ASD Using the Child Behavior Checklist 6–18. In: *Comprehensive Guide to Autism.* edn. Edited by Patel VB, Preedy VR, Martin CR. New York, NY: Springer New York; 2014: 2799–2812.

[24] Chua SY, Abd Rahman FN, Ratnasingam S. Problem behaviours and caregiver burden among children with Autism Spectrum Disorder in Kuching, Sarawak. Front Psychiatry 2023, 14. 10.3389/fpsyt.2023.1244164 PMC10642943 37965356

[25] Sepehrmanesh Z, Shahzeidi A, Mansournia MA, Ghaderi A, Ahmadvand A. Clinical and metabolic reaction to Probiotic supplement in children suffering attention-deficit hyperactivity disorder: a Randomized, Double-Blind, placebo-controlled experiment. Int Archives Health Sci 2021, 8(2).

[26] Ghanaatgar M, Taherzadeh S, Ariyanfar S, Razeghi Jahromi S, Martami F, Mahmoudi Gharaei J, Teimourpour A, Shahrivar Z. Probiotic supplement as an adjunctive therapy with Ritalin for treatment of attention-deficit hyperactivity disorder symptoms in children: a double-blind placebo-controlled randomized clinical trial. Nutr Food Sci. 2023;53(1):19–34.

[27] Lau-Zhu A, Fritz A, McLoughlin G. Overlaps and distinctions between attention deficit/hyperactivity disorder and autism spectrum disorder in young adulthood: systematic review and guiding framework for EEG-imaging research. Neurosci Biobehav Rev. 2019;96:93–115. 30367918 10.1016/j.neubiorev.2018.10.009 PMC6331660

[28] Pärtty A, Kalliomäki M, Wacklin P, Salminen S, Isolauri E. A possible link between early probiotic intervention and the risk of neuropsychiatric disorders later in childhood: a randomized trial. Pediatr Res. 2015;77(6):823–8. 25760553 10.1038/pr.2015.51

[29] Brett BE, Doumbia HOY, Koko BK, Koffi FK, Assa SE, Zahé KYAS, Kort R, Sybesma W, Reid G, de Weerth C. Normative cognition and the effects of a probiotic food intervention in first grade children in Côte d’Ivoire. Sci Rep. 2022;12(1):19491. 36376341 10.1038/s41598-022-23797-3 PMC9663712

[30] Wu CC, Wong LC, Hsu CJ, Yang CW, Tsai YC, Cheng FS, Hu HY, Lee WT. Randomized Controlled Trial of Probiotic PS128 in children with Tourette Syndrome. Nutrients 2021, 13(11). 10.3390/nu13113698 PMC8619307 34835954

[31] Alipour B, Homayouni-Rad A, Vaghef-Mehrabany E, Sharif SK, Vaghef-Mehrabany L, Asghari-Jafarabadi M, Nakhjavani MR, Mohtadi-Nia J. Effects of Lactobacillus casei supplementation on disease activity and inflammatory cytokines in rheumatoid arthritis patients: a randomized double-blind clinical trial. Int J Rheum Dis. 2014;17(5):519–27. 24673738 10.1111/1756-185X.12333

[32] Kullisaar T, Songisepp E, Mikelsaar M, Zilmer K, Vihalemm T, Zilmer M. Antioxidative probiotic fermented goats’ milk decreases oxidative stress-mediated atherogenicity in human subjects. Br J Nutr. 2003;90(2):449–56. 12908907 10.1079/bjn2003896

[33] Moher D, Shamseer L, Clarke M, Ghersi D, Liberati A, Petticrew M, Shekelle P, Stewart LA. Preferred reporting items for systematic review and meta-analysis protocols (PRISMA-P) 2015 statement. Syst Rev. 2015;4(1):1. 25554246 10.1186/2046-4053-4-1 PMC4320440

[34] McHugh ML. Interrater reliability: the kappa statistic. Biochem Med (Zagreb). 2012;22(3):276–82. 23092060 PMC3900052

[35] Higgins JP, Thomas J, Chandler J, Cumpston M, Li T, Page MJ, Welch VA. Cochrane handbook for systematic reviews of interventions. Wiley; 2019. 10.1002/14651858.ED000142 PMC10284251 31643080

[36] Guyatt GH, Oxman AD, Vist GE, Kunz R, Falck-Ytter Y, Alonso-Coello P, Schünemann HJ. GRADE: an emerging consensus on rating quality of evidence and strength of recommendations. BMJ. 2008;336(7650):924. 18436948 10.1136/bmj.39489.470347.AD PMC2335261

[37] Sanctuary MR, Kain JN, Chen SY, Kalanetra K, Lemay DG, Rose DR, Yang HT, Tancredi DA-O, German JB, Slupsky CM et al. Pilot study of probiotic/colostrum supplementation on gut function in children with autism and gastrointestinal symptoms. *PLoS One* 2019, 1(1932–6203 (Electronic)). 10.1371/journal.pone.0210064 PMC6326569 30625189

[38] Li YQ, Sun YH, Liang Yp Fau - Zhou F, Zhou F, Yang J, Jin SL. Effect of probiotics combined with applied behavior analysis in the treatment of children with autism spectrum disorder: a prospective randomized controlled trial. Zhongguo Dang Dai Er Ke Za Zhi. 2021;11(Print):1008–8830. 10.7499/j.issn.1008-8830.2108085 PMC8580031 34753541

[39] Bravo JA, Forsythe P, Chew MV, Escaravage E, Savignac HM, Dinan TG, Bienenstock J, Cryan JF. Ingestion of Lactobacillus strain regulates emotional behavior and central GABA receptor expression in a mouse via the vagus nerve. Proc Natl Acad Sci U S A. 2011;108(38):16050–5. 21876150 10.1073/pnas.1102999108 PMC3179073

[40] Jiang NM, Cowan M, Moonah SN, Petri WA Jr. The impact of systemic inflammation on Neurodevelopment. Trends Mol Med. 2018;24(9):794–804. 30006148 10.1016/j.molmed.2018.06.008 PMC6110951

[41] Davis RL. Chapter Eight - Neurodevelopment: Inflammation Matters. In: *Advances in Neurotoxicology. Volume 2*, edn. Edited by Aschner M, Costa LG: Academic Press; 2018: 227–264.

[42] Resta SC. Effects of probiotics and commensals on intestinal epithelial physiology: implications for nutrient handling. J Physiol. 2009;587(Pt 17):4169–74. 19596893 10.1113/jphysiol.2009.176370 PMC2754357

[43] Chang HY, Chen JH, Chang JH, Lin HC, Lin CY, Peng CC. Multiple strains probiotics appear to be the most effective probiotics in the prevention of necrotizing enterocolitis and mortality: an updated meta-analysis. PLoS ONE. 2017;12(2):e0171579. 28182644 10.1371/journal.pone.0171579 PMC5300201

[44] Slykerman RF, Kang J, Van Zyl N, Barthow C, Wickens K, Stanley T, Coomarasamy C, Purdie G, Murphy R, Crane J, et al. Effect of early probiotic supplementation on childhood cognition, behaviour and mood a randomised, placebo-controlled trial. Acta Paediatr. 2018;107(12):2172–8. 30246890 10.1111/apa.14590

[45] Firmansyah A, Dwipoerwantoro PG, Kadim M, Alatas S, Conus N, Lestarina L, Bouisset F, Steenhout P. Improved growth of toddlers fed a milk containing synbiotics. Asia Pac J Clin Nutr. 2011;20(1):69–76. 21393113

[46] Desbonnet L, Garrett L, Clarke G, Kiely B, Cryan JF, Dinan TG. Effects of the probiotic Bifidobacterium infantis in the maternal separation model of depression. Neuroscience. 2010;170(4):1179–88. 20696216 10.1016/j.neuroscience.2010.08.005

[47] Jiang H, Ling Z, Zhang Y, Mao H, Ma Z, Yin Y, Wang W, Tang W, Tan Z, Shi J, et al. Altered fecal microbiota composition in patients with major depressive disorder. Brain Behav Immun. 2015;48:186–94. 25882912 10.1016/j.bbi.2015.03.016

[48] Kato-Kataoka A, Nishida K, Takada M, Kawai M, Kikuchi-Hayakawa H, Suda K, Ishikawa H, Gondo Y, Shimizu K, Matsuki T, et al. Fermented milk containing Lactobacillus casei strain Shirota preserves the diversity of the gut microbiota and relieves abdominal dysfunction in Healthy Medical students exposed to academic stress. Appl Environ Microbiol. 2016;82(12):3649–58. 27208120 10.1128/AEM.04134-15 PMC4959178

[49] Guyonnet D, Chassany O, Ducrotte P, Picard C, Mouret M, Mercier CH, Matuchansky C. Effect of a fermented milk containing Bifidobacterium animalis DN-173 010 on the health-related quality of life and symptoms in irritable bowel syndrome in adults in primary care: a multicentre, randomized, double-blind, controlled trial. Aliment Pharmacol Ther. 2007;26(3):475–86. 17635382 10.1111/j.1365-2036.2007.03362.x

[50] Williams EA, Stimpson J, Wang D, Plummer S, Garaiova I, Barker ME, Corfe BM. Clinical trial: a multistrain probiotic preparation significantly reduces symptoms of irritable bowel syndrome in a double-blind placebo-controlled study. Aliment Pharmacol Ther. 2009;29(1):97–103. 18785988 10.1111/j.1365-2036.2008.03848.x

[51] Chao L, Liu C, Sutthawongwadee S, Li Y, Lv W, Chen W, Yu L, Zhou J, Guo A, Li Z, et al. Effects of Probiotics on depressive or anxiety variables in healthy participants under stress conditions or with a depressive or anxiety diagnosis: a Meta-analysis of Randomized controlled trials. Front Neurol. 2020;11:421. 32528399 10.3389/fneur.2020.00421 PMC7257376

[52] Mulay KV, Karthik SV. Managing constipation in children with ASD– a challenge worth tackling. Pediatr Neonatology. 2022;63(3):211–9. 10.1016/j.pedneo.2021.11.009 35190271

[53] Sayre CL, Yellepeddi VK, Job KM, Krepkova LV, Sherwin CMT, Enioutina EY. Current use of complementary and conventional medicine for treatment of pediatric patients with gastrointestinal disorders. Front Pharmacol. 2023;14:1051442. 36778015 10.3389/fphar.2023.1051442 PMC9911676

