# Supplementary material for: The therapeutic effects of probiotics on core and associated behavioral symptoms of autism spectrum disorders: a systematic review and meta-analysis
Source: Child Adolesc Psychiatry Ment Health. 2024 Dec 19;18:161. doi: 10.1186/s13034-024-00848-3 (PMC11660637; doi:10.1186/s13034-024-00848-3)

eTable 1. Applied keywords and the search results from each database

Database	Keywords	Filter	Date (yyyy/mm/dd)	Result
PubMed	(probiotics or psychobiotic or probio) and (autism or ASD or autism spectrum disorder or pervasive developmental disorder or Asperger syndrome)	Clinical trials	2023/11/21	19
Embase	(probiotics or psychobiotic or probio) and (autism or ASD or autism spectrum disorder or pervasive developmental disorder or Asperger syndrome)	RCT	2023/11/21	57
Cochrane CENTRAL	(probiotics or psychobiotic or probio) and (autism or ASD or autism spectrum disorder or pervasive developmental disorder or Asperger syndrome)	Trials	2023/11/21	74
ScienceDirect	probiotics and (Autism or ASD or Autism spectrum disorder)	Research articles	2023/11/21	163
Clinicaltrials.gov	Autism Spectrum Disorder and probiotics		2023/11/21	14

Abbreviations: NA, not applied; RCT, randomized controlled trial

eTable 2 Reasons for study exclusion

Reason	Number of excluded studies	References
Not targeted at ASD patients		[1-4]
Not using probiotics		[5-8]
Not Randomized clinical trials		[9]
No available data for analysis		[10-13]
Duplicated study		[14]

ADHD Attention deficit hyperactivity disorder

RCT randomized controlled trials

References

1. Yang, L.L., et al., *Effects of a Synbiotic on Plasma Immune Activity Markers and Short-Chain Fatty Acids in Children and Adults with ADHD-A Randomized Controlled Trial.* Nutrients, 2023. **15**(5).
2. Skott, E., et al., *Effects of a synbiotic on symptoms, and daily functioning in attention deficit hyperactivity disorder - A double-blind randomized controlled trial.* Brain Behav Immun, 2020. **89**: p. 9-19.
3. Severance, E.G., et al., *Probiotic normalization of Candida albicans in schizophrenia: A randomized, placebo-controlled, longitudinal pilot study.* Brain Behav Immun, 2017. **62**: p. 41-45.
4. Eghbalian, F., et al., *The Effect of Probiotics on Phototherapy for Bilirubin Reduction in Term Neonates: A Randomized Controlled Trial.*

Curr Pediatr Rev, 2023.
5. Grimaldi, R., et al., *A prebiotic intervention study in children with autism spectrum disorders (ASDs).* Microbiome, 2018. **6**(1): p. 133.
6. Kang, D.W., et al., *Microbiota Transfer Therapy alters gut ecosystem and improves gastrointestinal and autism symptoms: an open-label study.* Microbiome, 2017. **5**(1): p. 10.
7. Kang, D.W., et al., *Long-term benefit of Microbiota Transfer Therapy on autism symptoms and gut microbiota.* Sci Rep, 2019. **9**(1): p. 5821.
8. Wang, Y., et al., *Probiotics and fructo-oligosaccharide intervention modulate the microbiota-gut brain axis to improve autism spectrum reducing also the hyper-serotonergic state and the dopamine metabolism disorder.* Pharmacol Res, 2020. **157**: p. 104784.
9. Salmazo, G.F., et al., *Preliminary analysis of a clinical trial of children with autism spectrum disorder treated with DHA-rich marine Schizochytrium sp. oil and multi-vitamin/mineral complex.* Research in Autism Spectrum Disorders, 2023. **109**: p. 102282.
10. Zhang, L., et al., *The role of probiotics in children with autism spectrum disorders: A study protocol for a randomised controlled trial.* PLoS One, 2022. **17**(2): p. e0263109.
11. Pärtty, A., et al., *A possible link between early probiotic intervention and the risk of neuropsychiatric disorders later in childhood: a randomized trial.* Pediatr Res, 2015. **77**(6): p. 823-8.
12. Guidetti, C., et al., *Randomized Double-Blind Crossover Study for Evaluating a Probiotic Mixture on Gastrointestinal and Behavioral Symptoms of Autistic Children.* J Clin Med, 2022. **11**(18).
13. Niu, M., et al., *Characterization of Intestinal Microbiota and Probiotics Treatment in Children With Autism Spectrum Disorders in China.* Front Neurol, 2019. **10**: p. 1084.
14. Sherman, H.T., et al., *Carbon monoxide (CO) correlates with symptom severity, autoimmunity, and responses to probiotics treatment in a cohort of children with autism spectrum disorder (ASD): a post-hoc analysis of a randomized controlled trial.* BMC Psychiatry, 2022. **22**(1): p. 536.

eTable 3. Grading of Recommendations Assessments, Development and Evaluation (GRADE) assessment of the strength of evidence for standard weighted meta-analysis

Outcome	Design	Risk of bias	Indirectness	Inconsistency	Imprecision	Publication bias	Grade Quality
Overall behavioral symptoms of ASD	RCT x10	No Serious	No indirectness	No serious inconsistency	Serious imprecision	Undetected	⊕⊕⊕O[4]
Restricted repetitive behaviors	RCT x7	No Serious	No indirectness	No serious inconsistency	Serious imprecision	Undetected	⊕⊕⊕O[4]
Social behaviors	RCT x 8	No Serious	No indirectness	No serious inconsistency	Serious imprecision	Undetected	⊕⊕⊕O[4]
Communication	RCT x 6	No Serious	No indirectness	No serious inconsistency	Serious imprecision	Undetected	⊕⊕OO[4*]
Irritability/aggression	RCT x7	No Serious	Serious indirectness	No serious inconsistency	Serious imprecision	Undetected	⊕⊕OO[4]
Hyperactivity/impulsivity	RCT x7	No Serious	Serious indirectness	No serious inconsistency	Serious imprecision	Undetected	⊕⊕OO[4]
Parental stress	RCT x3	No Serious	Serious indirectness	No serious inconsistency	Serious imprecision	Undetected	⊕OOO[4*]
Inattention	RCT x2	No Serious	Serious indirectness	No serious inconsistency	Serious imprecision	Undetected	⊕OOO[4*]

Anxiety	RCT x3	No Serious	Serious indirectness	No serious inconsistency	Serious imprecision	Undetected	⊕OOO[4*]
Adaptation	RCT x3	No Serious	Serious indirectness	No serious inconsistency	Serious imprecision	Undetected	⊕OOO[4*]

*Risk of bias was estimated using Cochrane risk of bias, studies were classified as having low risk of bias if none of the domains above was rated as high risk of bias and three or less were rated as unclear risk; moderate if one was rated as high risk of bias or none was rated as high risk of bias but four or more were rated as unclear risk, and all other cases were assumed to pertain to high risk of bias.

Down-graded due to: [1] risk of bias, [2] indirectness, [3.] Inconsistency, [4.] Imprecision, [5.] publication bias

[4*] Down-graded two levels due to very low sample sizes

Abbreviation: ASD autism spectrum disorder

GRADE Working Group grades of evidence:

-**High certainty**: We are very confident that the true effect lies close to that of the estimate of the effect

-**Moderate certainty**: We are moderately confident in the effect estimate: The true effect is likely to be close to the estimate of the effect, but there is a possibility that it is substantially different.

-**Low certainty**: Our confidence in the effect estimate is limited: The true effect may be substantially different from the estimate of the effect.

-**Very low certainty**: We have very little confidence in the effect estimate: The true effect is likely to be substantially different from the estimate of effect.

eFigure 1. Funnel plot – overall behavioral symptoms

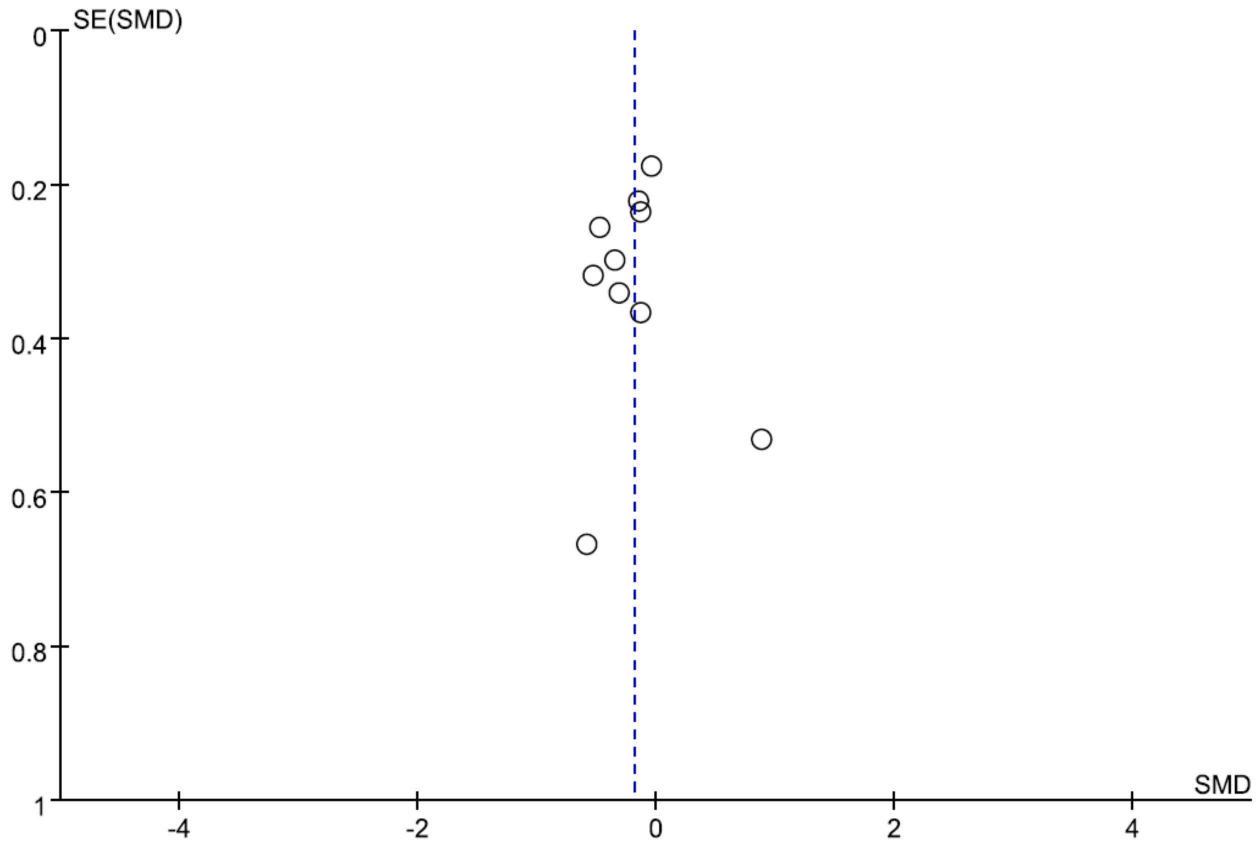

eFigure 2 Forest plot of effect size for comparing the difference in the overall behavioral symptoms of autism spectrum disorder between probiotics and control groups in subgroups of studies using probiotics as supplementation

	Probiotics			Placebo				Std. Mean Difference	
Study or Subgroup	Mean	SD	Total	Mean	SD	Total	Weight	IV, Random, 95% CI	Year
**3.1.1 Supplementation**									
Emmanuel (NCT03369431)	-12.122	20.914	64	-11.427	20.306	64	27.8%	-0.03 [-0.38, 0.31]	
Liu (2019)	15.11	9.14	36	16.36	10.16	35	15.4%	-0.13 [-0.59, 0.34]	2019
Arnold (2019)	75.67	11.021	6	82.5	10.376	4	2.0%	-0.57 [-1.88, 0.73]	2019
Santocchi (2020)	6.19	1.56	31	7	1.8	32	13.3%	-0.47 [-0.98, 0.03]	2020
Kong (2021)	6.67	26	18	15	26.75	17	7.5%	-0.31 [-0.98, 0.36]	2021
Liu (2023)	-2.95	5.58	41	-2.05	6.59	41	17.8%	-0.15 [-0.58, 0.29]	2023
Schmitt (2023)	3.87	0.5	15	3.93	0.46	15	6.5%	-0.12 [-0.84, 0.59]	2023
Billeci (2023)	6.3	1.6	26	6.9	1.9	20	9.7%	-0.34 [-0.93, 0.25]	2023
**Subtotal (95% CI)**			**237**			**228**	**100.0%**	**-0.19 [-0.38, -0.01]**	
Heterogeneity: $Tau^2 = 0.00$; $Chi^2 = 2.86$, df = 7 (P = 0.90); $I^2 = 0\%$									
Test for overall effect: Z = 2.07 (P = 0.04)									
**Total (95% CI)**			**237**			**228**	**100.0%**	**-0.19 [-0.38, -0.01]**	
Heterogeneity: $Tau^2 = 0.00$; $Chi^2 = 2.86$, df = 7 (P = 0.90); $I^2 = 0\%$									
Test for overall effect: Z = 2.07 (P = 0.04)									
Test for subgroup differences: Not applicable									

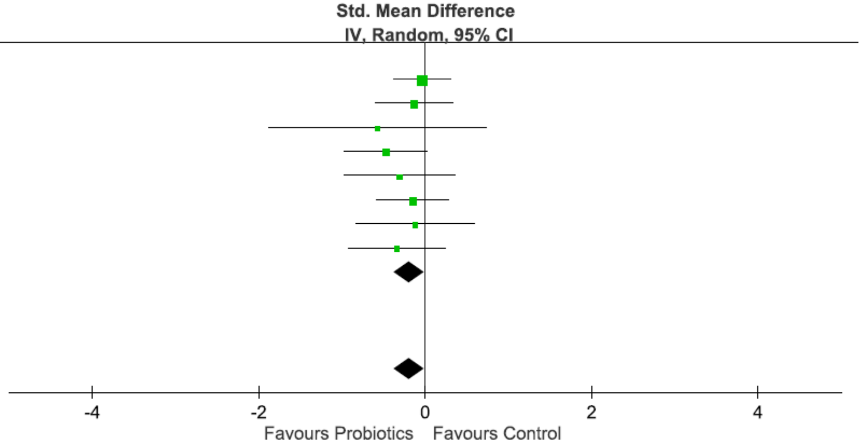

eFigure 3. Funnel plot –restricted repetitive behaviors

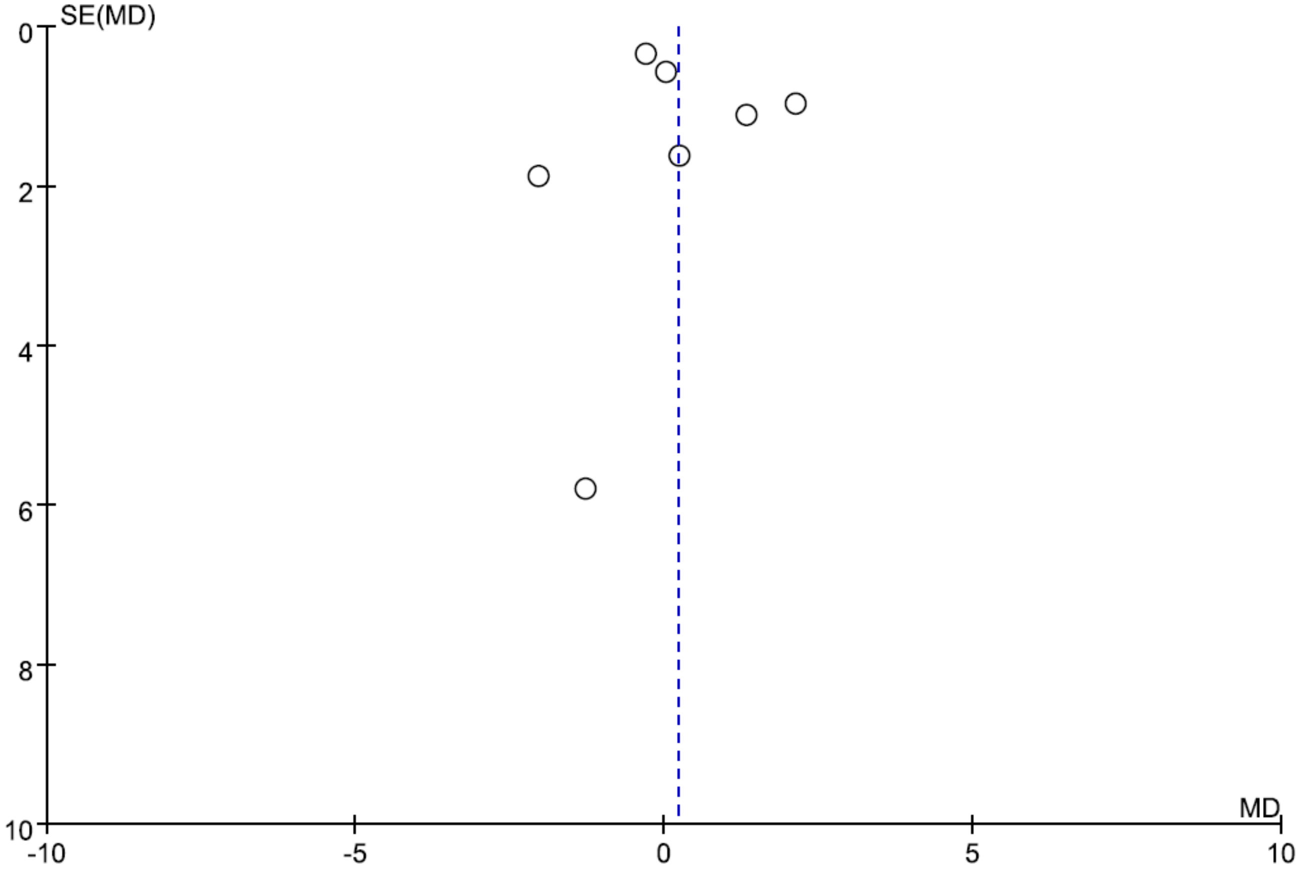

eFigure 4. Funnel plot – social behaviors

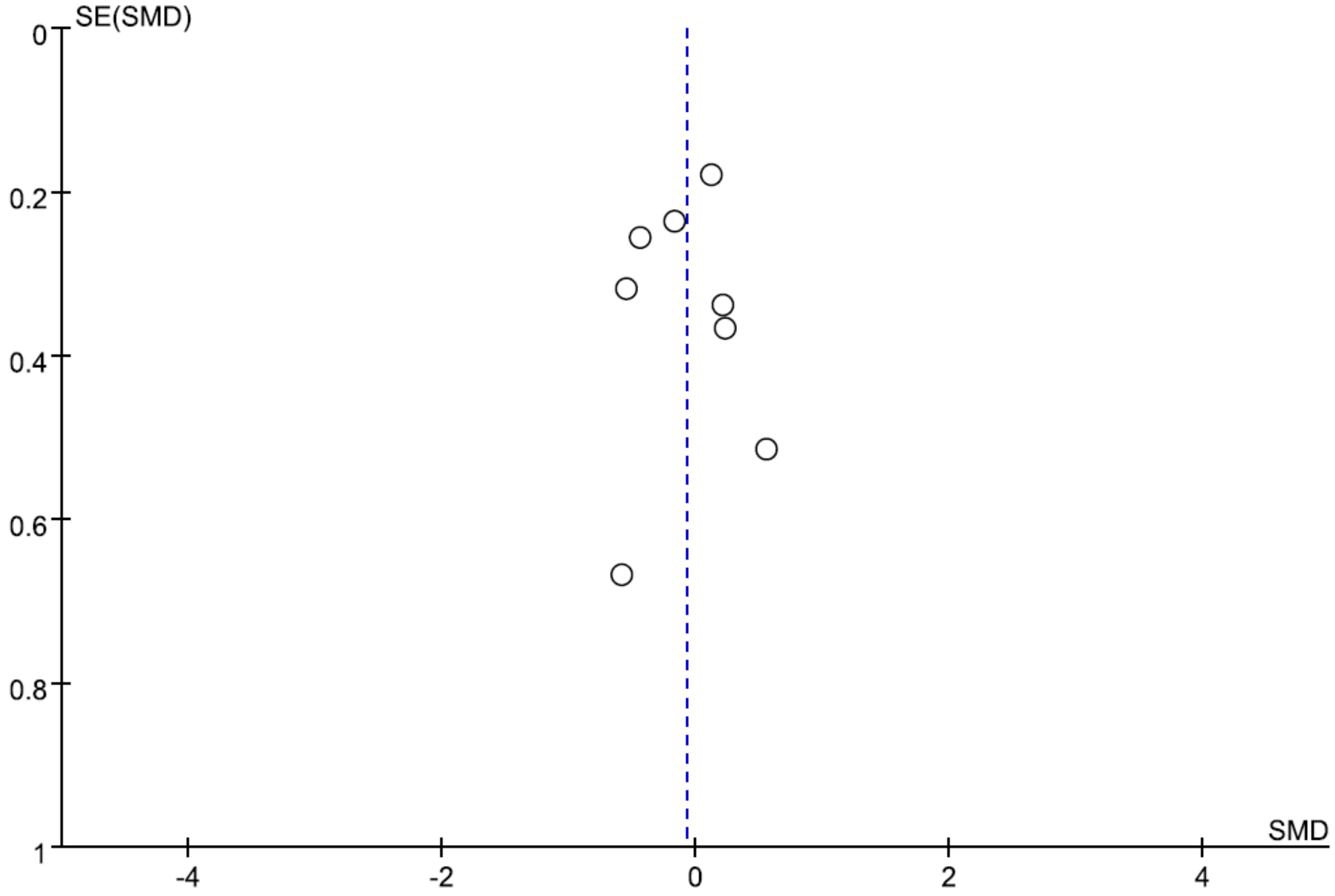

eFigure 5. Funnel plot –communication

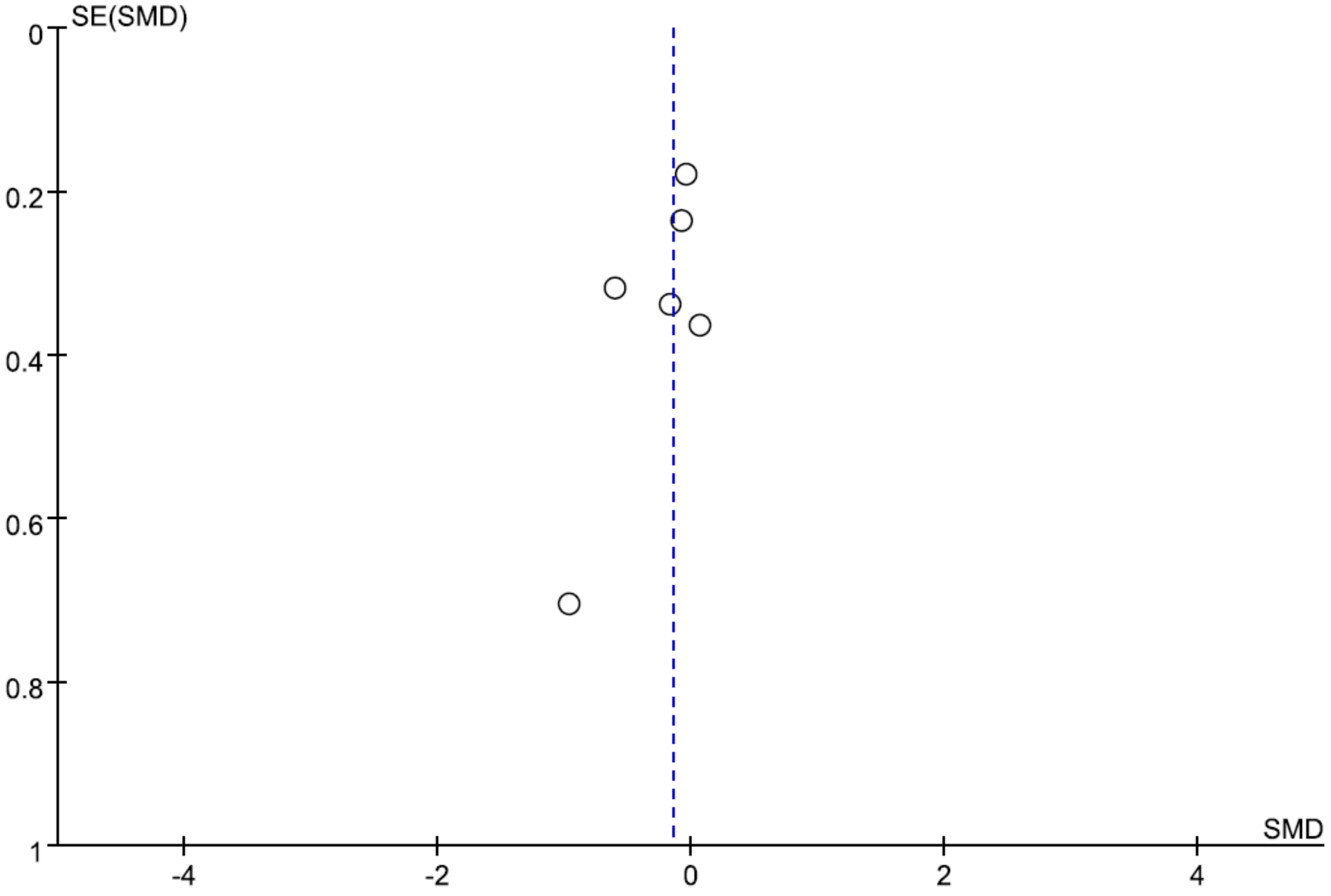

eFigure 6. Forest plot of effect size for comparing the difference in irritability/aggression between probiotics and placebo groups

Study or Subgroup	Probiotics Mean	Probiotics SD	Probiotics Total	Placebo Mean	Placebo SD	Placebo Total	Weight	Std. Mean Difference IV, Random, 95% CI	Year
Emmanuel (NCT03369431)	-3	8.66	62	-3.05	8.39	62	34.1%	0.01 [-0.35, 0.36]	
Sanctuary (2018)	-2.125	7.754	8	-6.375	3.998	8	4.1%	0.65 [-0.36, 1.67]	2018
Liu (2019)	8.06	7.02	36	7.94	6.02	35	19.5%	0.02 [-0.45, 0.48]	2019
Arnold (2019)	14.17	9.065	6	21.25	12.42	4	2.5%	-0.61 [-1.92, 0.70]	2019
Kong (2021)	-0.92	6.2	18	3.45	6.67	17	9.1%	-0.66 [-1.35, 0.02]	2021
Liu (2023)	-1.7	5.32	41	-0.77	9.95	41	22.5%	-0.12 [-0.55, 0.32]	2023
Schmitt (2023)	2.4	3.49	15	3.13	4.22	15	8.2%	-0.18 [-0.90, 0.53]	2023
**Total (95% CI)**			**186**			**182**	**100.0%**	**-0.08 [-0.29, 0.12]**	

Heterogeneity: Tau² = 0.00; Chi² = 5.94, df = 6 (P = 0.43); I² = 0%

Test for overall effect: Z = 0.80 (P = 0.42)

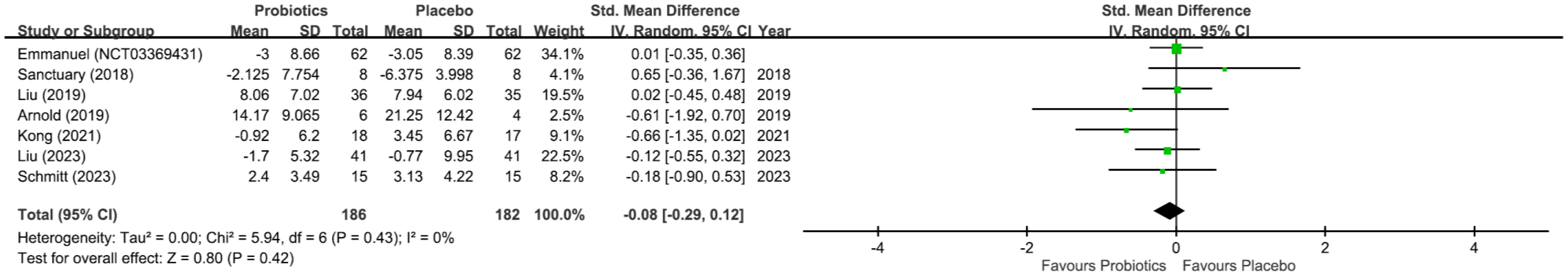

eFigure 7. Forest plot of effect size for comparing the difference in hyperactivity/impulsivity between probiotics and placebo groups

	Probiotics			Placebo				Std. Mean Difference	
Study or Subgroup	Mean	SD	Total	Mean	SD	Total	Weight	IV, Random, 95% CI	Year
Emmanuel (NCT03369431)	-2.42	7.43	62	-2.16	7.04	62	34.0%	-0.04 [-0.39, 0.32]	
Sanctuary (2018)	-2.5	5.014	8	-6.25	4.743	8	4.0%	0.73 [-0.30, 1.75]	2018
Liu (2019)	9.65	5.23	36	10.25	6.42	35	19.4%	-0.10 [-0.57, 0.36]	2019
Arnold (2019)	7	7.266	6	8.25	9.946	4	2.6%	-0.13 [-1.40, 1.13]	2019
Kong (2021)	1.17	6.64	18	3.18	4.38	17	9.4%	-0.35 [-1.02, 0.32]	2021
Schmitt (2023)	7.67	5.96	15	6.07	5.87	15	8.1%	0.26 [-0.46, 0.98]	2023
Liu (2023)	-0.11	4.29	41	0.56	4.84	41	22.4%	-0.15 [-0.58, 0.29]	2023
**Total (95% CI)**			**186**			**182**	**100.0%**	**-0.05 [-0.26, 0.16]**	

Heterogeneity: Tau² = 0.00; Chi² = 3.96, df = 6 (P = 0.68); I² = 0%
Test for overall effect: Z = 0.48 (P = 0.63)

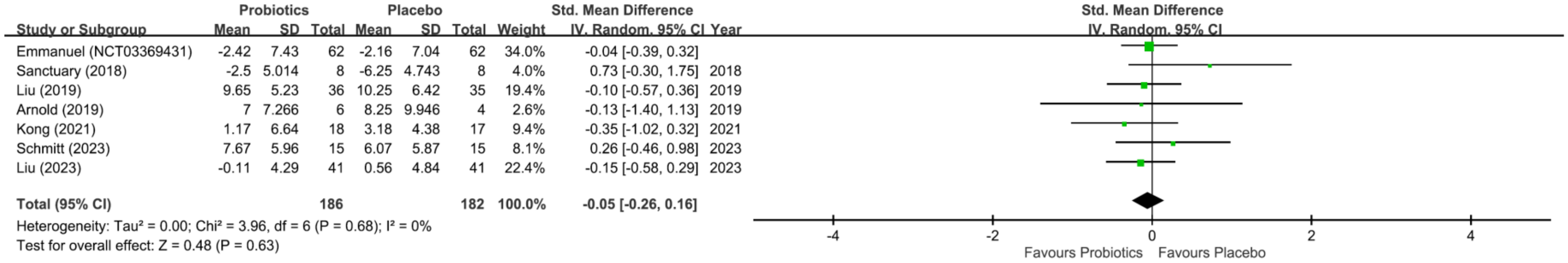

eFigure 8. Forest plot of effect size for comparing the difference in parental stress between probiotics and placebo groups

Study or Subgroup	Probiotics Mean	SD	Total	Placebo Mean	SD	Total	Weight	Std. Mean Difference IV, Random, 95% CI	Year
Emmanuel (NCT03369431)	-3.73	7.42	64	-3.11	6.98	64	64.0%	-0.09 [-0.43, 0.26]	
Arnold (2019)	96.33	19.315	6	105.25	22.187	4	4.7%	-0.39 [-1.68, 0.89]	2019
Santocchi (2020)	66.62	31.15	31	61.03	32.58	32	31.4%	0.17 [-0.32, 0.67]	2020
**Total (95% CI)**			**101**			**100**	**100.0%**	**-0.02 [-0.30, 0.26]**	

Heterogeneity: $Tau^2 = 0.00$; $Chi^2 = 1.05$, df = 2 (P = 0.59); $I^2 = 0\%$

Test for overall effect: Z = 0.13 (P = 0.89)

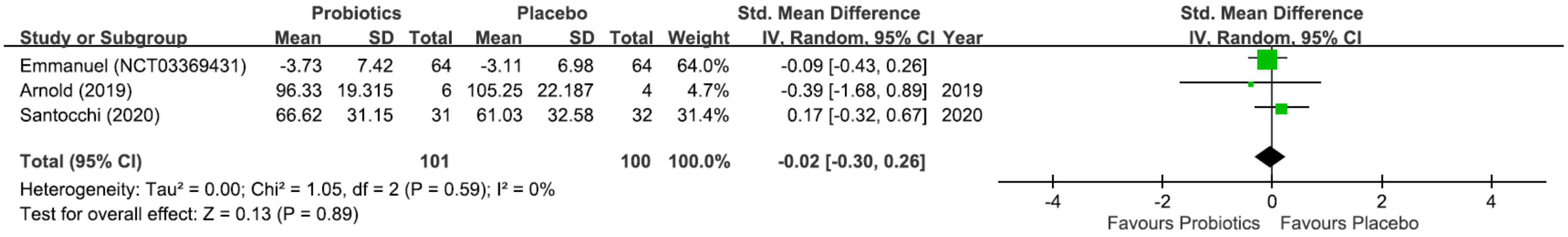

eFigure 9. Forest plot of effect size for comparing the difference in inattention between probiotics and placebo groups

	Probiotics			Placebo				Std. Mean Difference	
Study or Subgroup	Mean	SD	Total	Mean	SD	Total	Weight	IV, Random, 95% CI	Year
Liu (2019)	14.39	5.91	36	15.35	5.48	35	46.7%	-0.17 [-0.63, 0.30]	2019
Liu (2023)	-0.92	4.19	41	0.72	5.39	41	53.3%	-0.34 [-0.77, 0.10]	2023
**Total (95% CI)**			**77**			**76**	**100.0%**	**-0.26 [-0.58, 0.06]**	

Heterogeneity: $Tau^2 = 0.00$; $Chi^2 = 0.27$, $df = 1$ ($P = 0.60$); $I^2 = 0\%$
Test for overall effect: $Z = 1.58$ ($P = 0.11$)

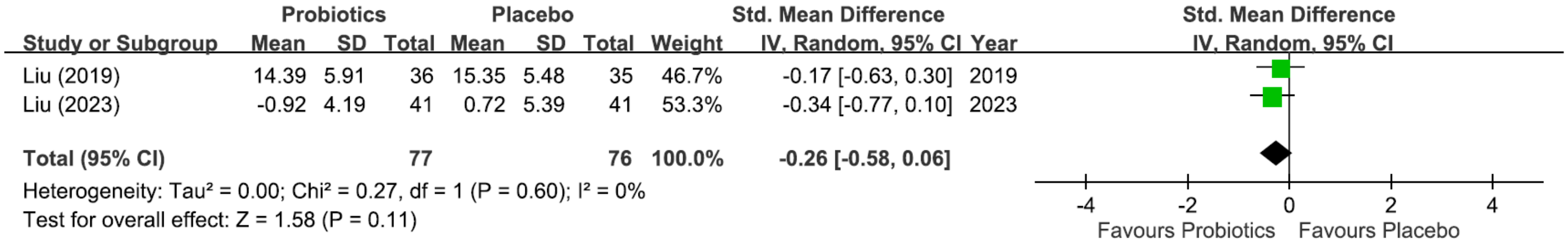

eFigure 10. Funnel plot – irritability/aggression

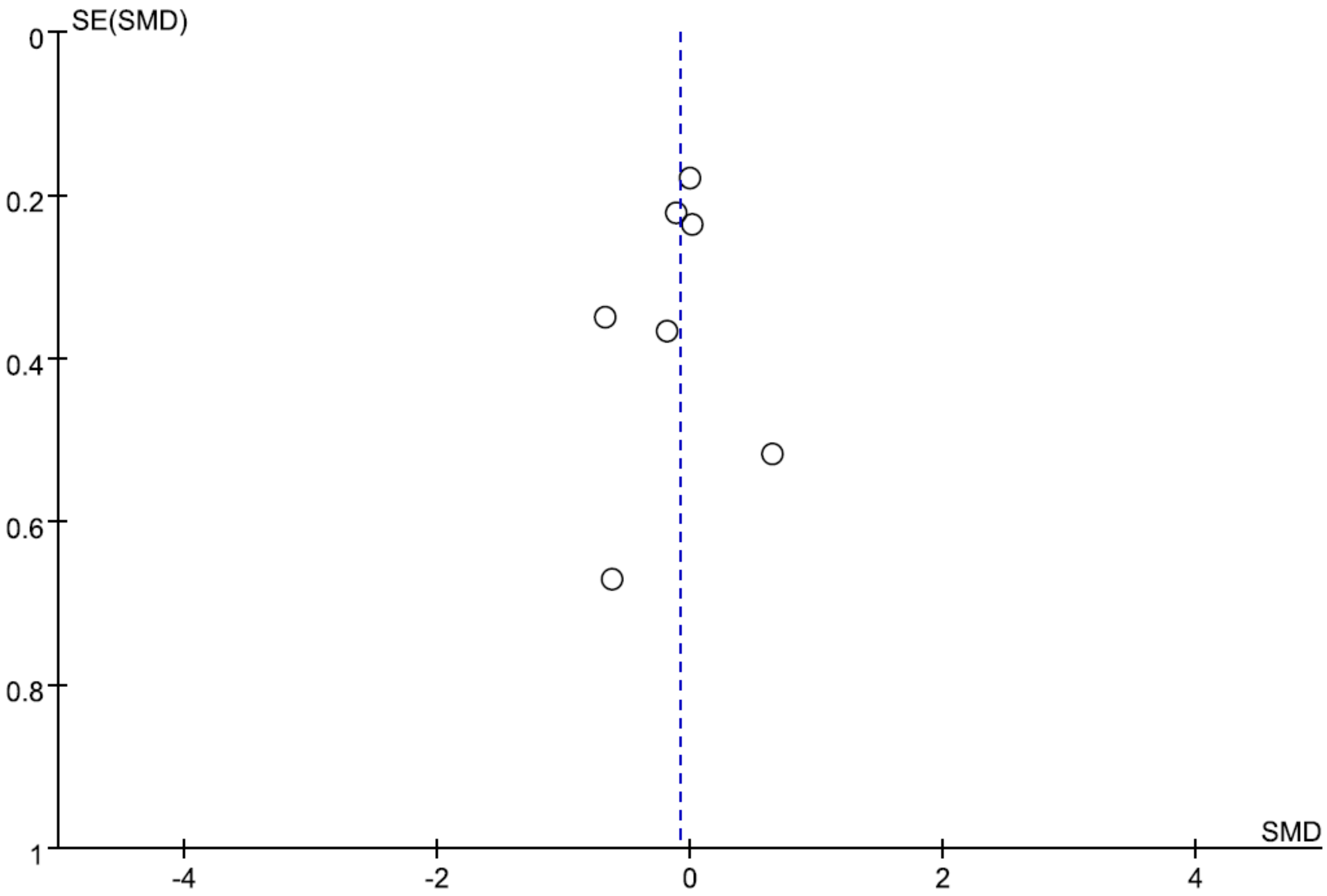

eFigure 11. Funnel plot – hyperactivity/impulsivity

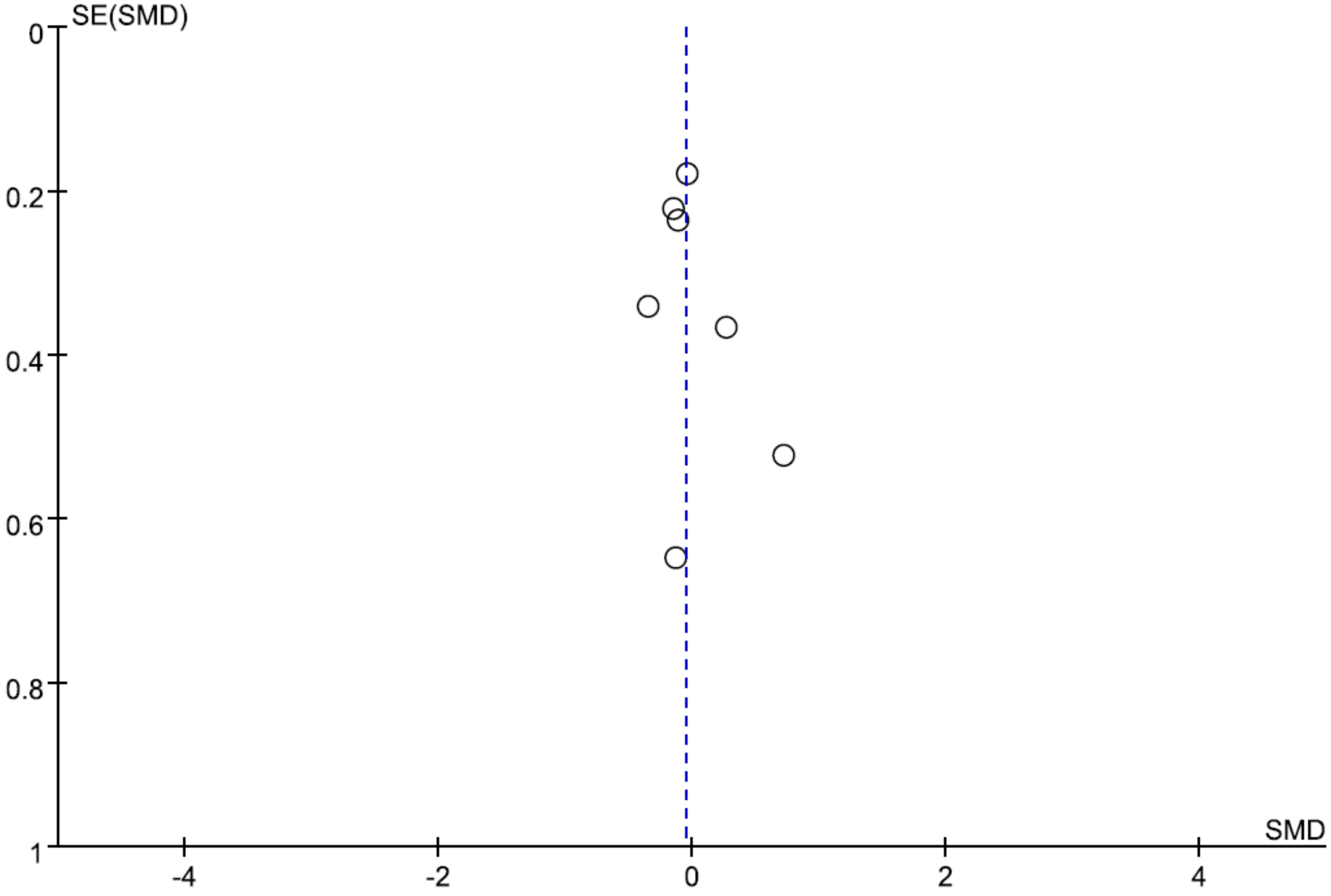

eFigure 12. Funnel plot – parental stress

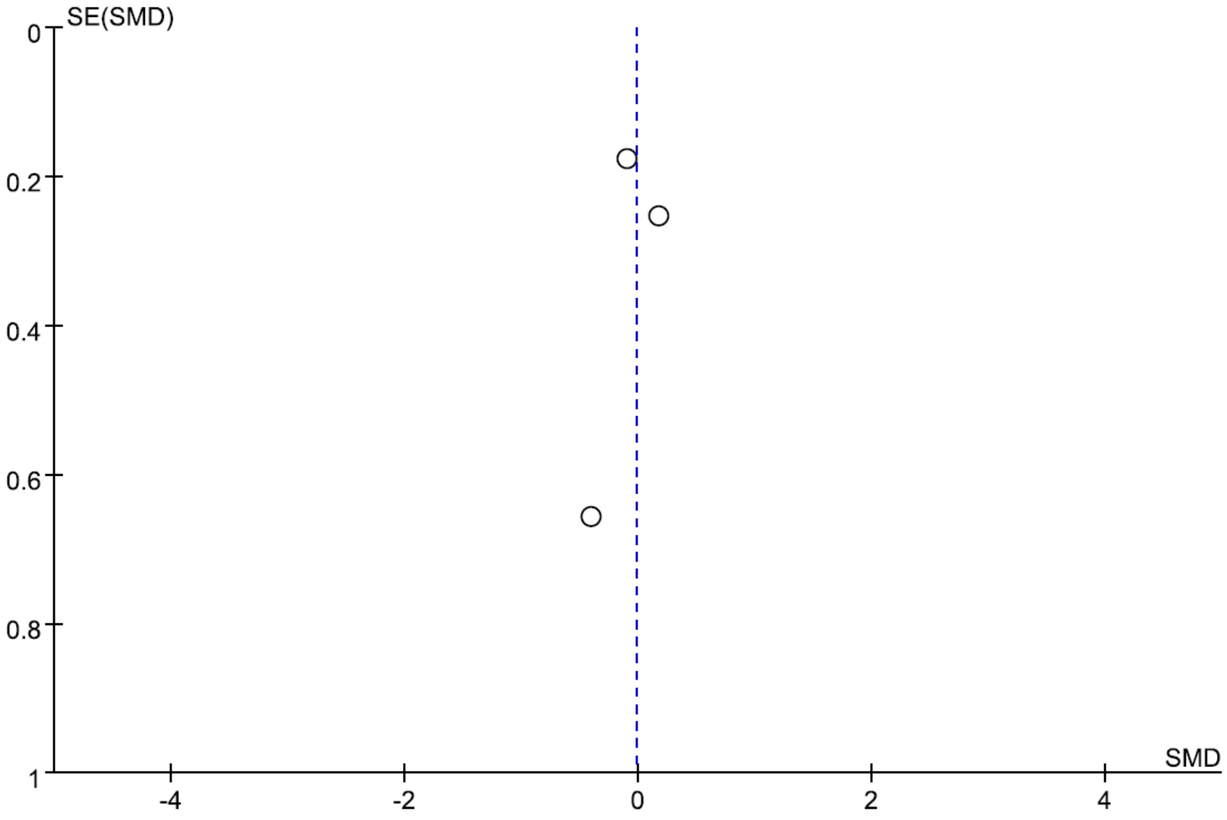

eFigure 13. Funnel plot – inattention

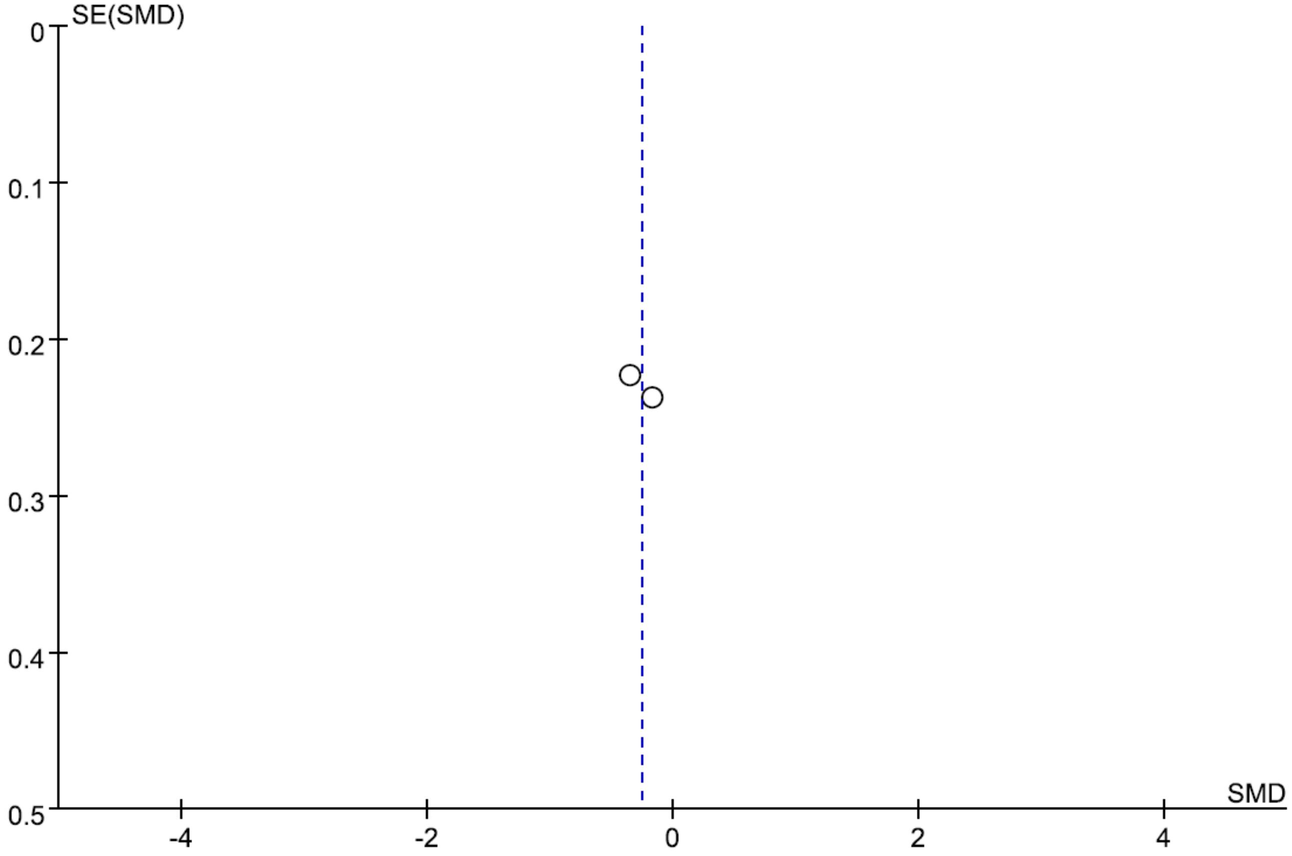

eFigure 14. Forest plot of effect size for comparing the difference in anxiety between probiotics and placebo groups

	Probiotics			Placebo				Std. Mean Difference	
Study or Subgroup	Mean	SD	Total	Mean	SD	Total	Weight	IV, Random, 95% CI	Year
Liu (2019)	5.63	4.34	36	5.84	4.41	35	44.4%	-0.05 [-0.51, 0.42]	2019
Arnold (2019)	28.67	13.322	6	34.75	19.12	4	5.9%	-0.35 [-1.63, 0.93]	2019
Liu (2023)	-2.19	5.95	41	1.36	8.04	41	49.7%	-0.50 [-0.94, -0.06]	2023
**Total (95% CI)**			**83**			**80**	**100.0%**	**-0.29 [-0.60, 0.02]**	

Heterogeneity: Tau² = 0.00; Chi² = 1.90, df = 2 (P = 0.39); I² = 0%

Test for overall effect: Z = 1.82 (P = 0.07)

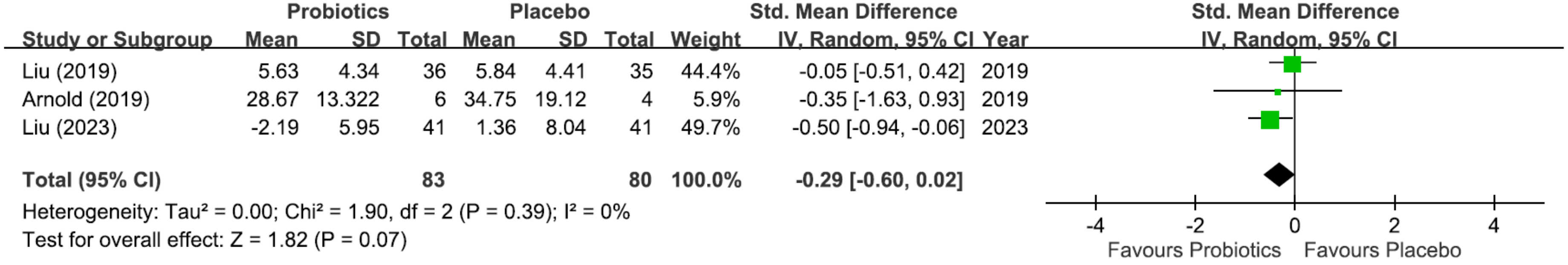

eFigure 15. Funnel plot – anxiety

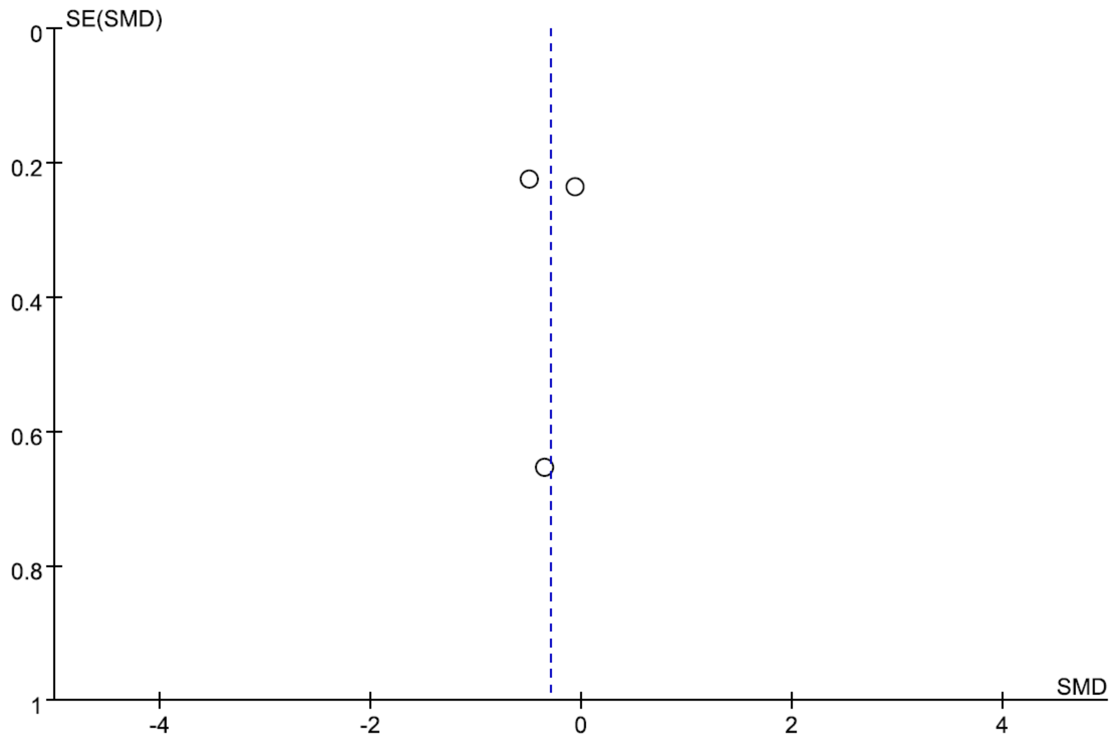

eFigure 16. Forest plot of effect size for comparing the difference in adaptation between probiotics and placebo groups

	Probiotics			Placebo				Std. Mean Difference	
Study or Subgroup	Mean	SD	Total	Mean	SD	Total	Weight	IV, Random, 95% CI	Year
Santocchi (2020)	67.39	22.29	31	59.72	16.38	32	45.5%	0.39 [-0.11, 0.89]	2020
Schmitt (2023)	4.87	7.98	15	1.05	7.98	15	21.5%	0.47 [-0.26, 1.19]	2023
Billeci (2023)	67.5	21.4	26	61.9	16.3	20	33.0%	0.28 [-0.30, 0.87]	2023
**Total (95% CI)**			**72**			**67**	**100.0%**	**0.37 [0.03, 0.71]**	

Heterogeneity: Tau² = 0.00; Chi² = 0.15, df = 2 (P = 0.93); I² = 0%
Test for overall effect: Z = 2.16 (P = 0.03)

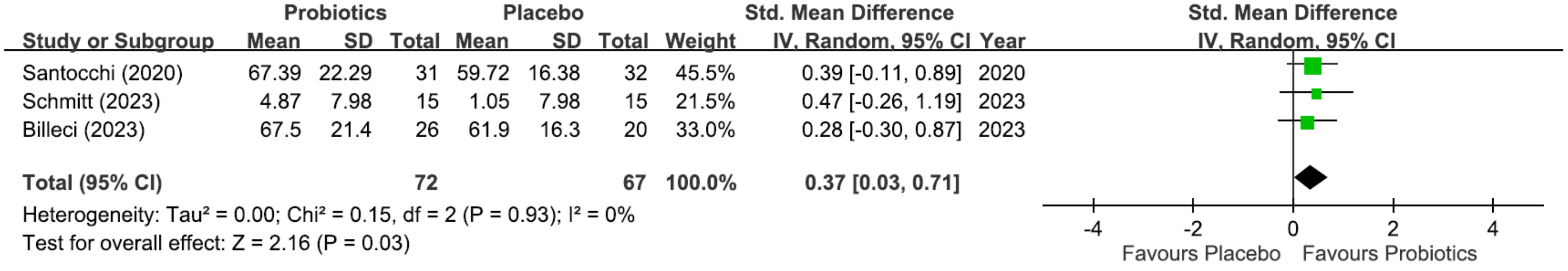

eFigure 17. Funnel plot – adaptation

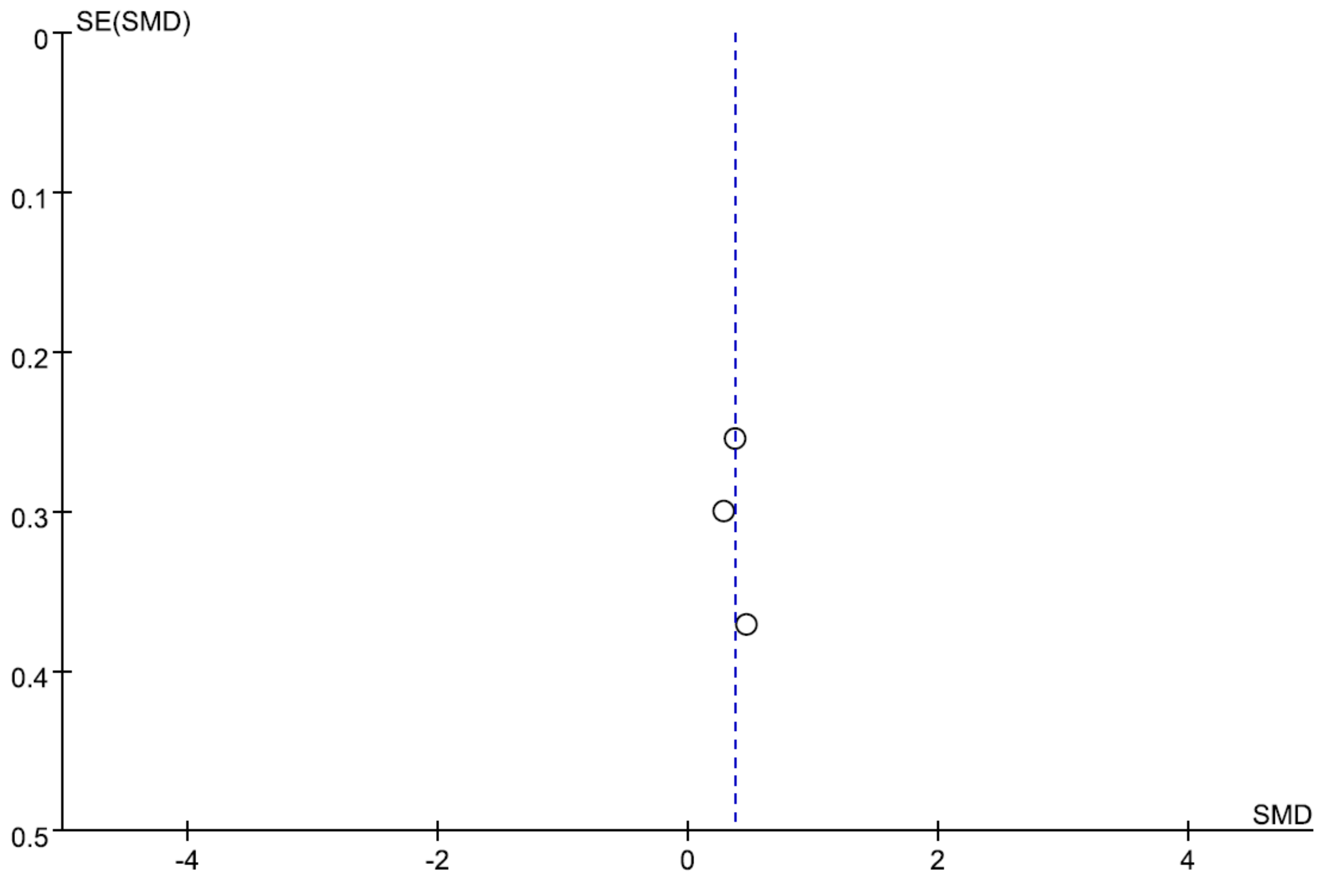

Supplement: Supplementary file 1 — Supplementary Material 1 [file 13034_2024_848_MOESM1_ESM.pdf]
